# Synthetic Strategy and Anti-Tumor Activities of Macrocyclic Scaffolds Based on 4-Hydroxyproline

**DOI:** 10.3390/molecules21020212

**Published:** 2016-02-15

**Authors:** Guorui Cao, Kun Yang, Yue Li, Longjiang Huang, Dawei Teng

**Affiliations:** 1 College of Chemical Engineering, Qingdao University of Science and Technology, Qingdao 266042, China; llxx.love@163.com (G.C.); yk060606@126.com (K.Y.); a4931778@163.com (Y.L.); routechem@163.com (L.H.); 2 State Key Laboratory of Bioactive Substance and Function of Natural Medicines, Institute of Materia Medica, Chinese Academy of Medical Sciences and Peking Union Medical College, Beijing 100050, China

**Keywords:** macrocycle, 4-hydroxyproline, azide-alkyne cycloaddition, Mitsunobu reaction, amide formation, anti-tumor activity

## Abstract

A series of novel 13- to 15-member hydroxyproline-based macrocycles, which contain alkyl-alkyl ether and alkyl-aryl ether moieties, have been synthesized by the strategy of macrocyclization utilising azide-alkyne cycloaddition, Mitsunobu protocol and amide formation. Their anti-tumor activities towards A549, MDA-MB-231 and Hep G2 cells were screened *in vitro* by an MTT assay. The results indicated that 13-member macrocycle **33** containing alkene chain showed the best results, exhibiting the highest inhibitory effects towards lung cancer cell line A549, which was higher than that of the reference cisplatin (IC_50_ value = 2.55 µmol/L).

## 1. Introduction

Macrocycles are commonly found in bioactive natural products and used as valuable source of bioactive molecules in drug discovery. They can demonstrate drug-like physicochemical and pharmacokinetic properties such as good solubility, lipophilicity, metabolite stability and bioavailability [1,2]. Macrocyclic structures could provide a compromise between structural pre-organization and sufficient flexibility to mould to a target protein surface and maximize binding interactions [3]. They also have a favorable impact on other essential properties required for drugs, such as membrane permeability, metabolic stability, increased potencies, better receptor selectivity and overall pharmacokinetics [4,5,6,7]. The aryl ether moiety is a common structural motif of many bioactive macrocyclic natural products such as vancomycin family of antibiotics [8,9,10,11], noncompetitive ACE inhibitor K-13 [12,13], piperazinomycin [14,15,16], and serine-based macrocycles representing β-turn mimetics [17,18,19,20]. Some hydroxyproline-based macrocycles have been introduced into important drugs, such as ACE inhibitor zizyphine [21,22,23], alkaloid paliurine E [24], and the echinocandin family including anidulafungin, caspofungin, and micafungin [25,26,27,28]. Recently, HCV NS3 protease inhibitors vaniprevir (MK-7009) [29,30], ITMN-191 [31], and BILN 2601 [32] were advanced into clinical development.

**Figure 1:**
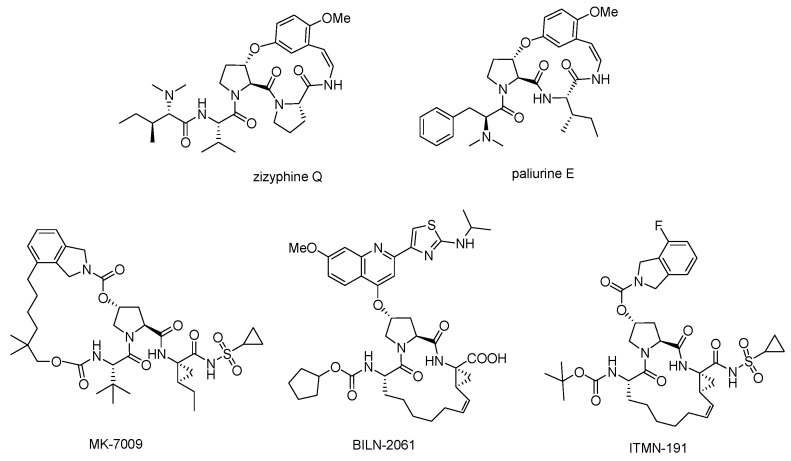
Hydroxyproline-containing nature products and drugs.

**Figure 2:**
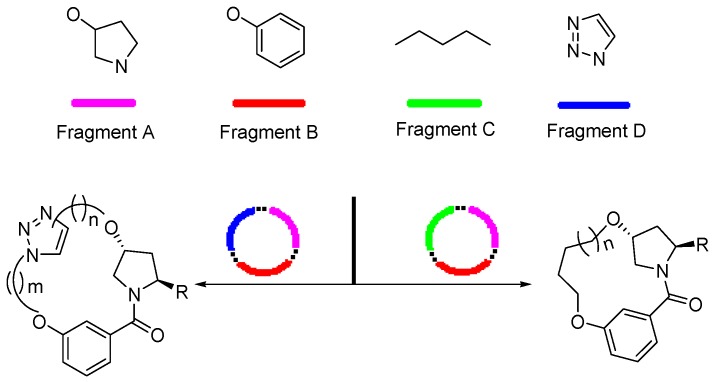
Target macrocyclic structures.

Inspired by the numerous aryl-ether moieties found in nature and hydroxyproline-based macrocycles discovered in drugs (Figure 1), we designed macrocyclic structures that incorporated aryl-ether and hydroxyproline fragments. Furthermore, another two fragments were introduced into the target structures to provide macrocycles of suitable size and conformation (Figure 2). It is well known that macrocycle size is carried out prior to the synthesis of macrocycles—large enough not to be strained and small enough to avoid clashes with the protein. As part of our previous efforts on exploring biologically important heterocyclic compounds and natural products [33,34,35], we herein report the successful synthesis of a collection of 13- to 15-member macrocyclic scaffolds, which incorporate 1,3-benzene rings and hydroxyproline, and evaluate their anti-tumor activities toward human tumor cell lines A549, MDA-MB-231 and Hep G2.

## 2. Results and Discussion

### 2.1. Chemistry

**Scheme 1:**
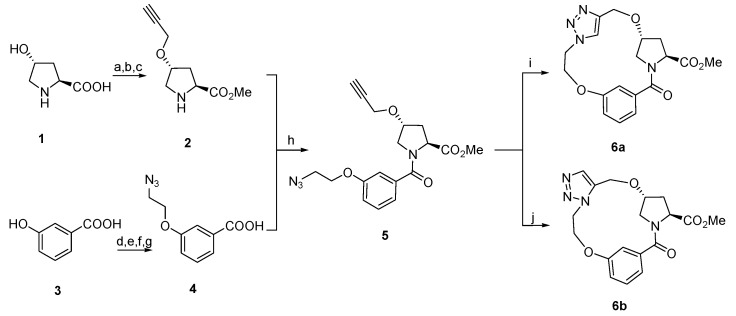
Macrocyclization with azide-alkyne cycloaddition. *Reagents and conditions*: (a) (i) SOCl_2_, MeOH, reflux, 5 h; (ii) (Boc)_2_O, MeOH, 0 °C–r.t., 2 h, 85%; (b) 3-Bromo-propyne, NaH, DMF, 0 °C–r.t., overnight, 37%; (c) 3M HCl/EtOAc, r.t., 2 h, 95%; (d) SOCl_2_, MeOH, reflux, 5 h, 96%; (e) 1-Bromo-2-chloro-ethane, NaH, DMF, 0 °C–r.t., overnight, 34%; (f) NaN_3_, DMF, 50 °C, 2 h, 85%; (g) NaOH, MeOH/H_2_O, 3 h, 92%; (h) HOBt, EDCI, Et_3_N, CH_2_Cl_2_, 90%; (i) CuI, toluene, reflux, 2 h, 43%; (j) [Cp*RuCl]_4_, toluene, 80 °C, 4 h, 42%.

Of all the macrocyclization methods, Huisgen 1,3-dipolar cycloaddition [36] of azides and alkynes is proposed to construct a triazole-linker to cyclize the macrocyclic ring. Apparently, it is straightforward and the ring size could be easily manipulated. Under thermal conditions, the reactions produce a mixture of 1,4- and 1,5-disubstituted 1,2,3-triazole regioisomers. This was overcome by the discovery of Cu(I)-catalyzed azide-alkyne cycloaddition (CuAAC) [37,38,39,40,41,42] and ruthenium-catalyzed azide-alkyne cycloaddition (RuAAC) [43,44,45], which could lead regioselectively to 1,4-disubstituted and 1,5-disubstituted 1,2,3-triazoles. The synthesis was illustrated in Scheme 1. *trans*-4-Hydroxy-l-proline (**1**) was sequentially esterified, *N*-protected with a Boc group, alkylated with 3-bromopropyne, and *N*-deprotected under acidic conditions to afford alkyne **2**. 3-Hydroxybenzoic acid (**3**) was sequentially methylated with thionyl chloride in methanol, alkylated with 1-bromo-2-chloroethane, substituted by sodium azide, and hydrolysed to afford azide **4**. Reaction of alkyne **2** with azide **4** affords **5** under amide formation conditions (Scheme 1).

With compound **5** in hand, the intramolecular 1,3-dipolar cycloaddition was conducted under various conditions (Table 1). Under thermal conditions, a mixture of 1,4- and 1,5-disubstituted triazole linkers was obtained in a ratio of 5:1 after refluxing in toluene (Entry 1). Under CuAAC conditions, the reaction led exclusively to the 1,4-disubstituted triazole linker **6a** in 55% yield (Entry 2). Under RuAAC conditions, the cyclization failed employing Cp*RuCl(COD) as catalyst, possibly due to the thermal instability of the catalyst [46] (Entries 3, 4). After several attempts, we found that the macrocycle with 1,5-disubstituted triazole linker was formed using Cp*RuCl(PPh_3_)_2_ as catalyst (Entry 5). However, the product was hard to isolate from the phosphine oxide formed in the reaction [47]. Improved result was achieved with [Cp*RuCl]_4_ as catalyst in toluene at 80 °C in 48% yield (Entry 6). Compared to 15-membered macrocycle **6a**, 14-membered product **6b** was obtained in lower yield and the reaction needed longer time (Entries 2 and 7).

**Table 1:** Macrocyclization with azide-alkyne cycloadditon ^a^.

Entry	Catalyst	Temperature (°C)	Time (h)	Ring Size	Yield (%) ^b^	Ratio (1,4-:1,5-)
1	Thermal	110	24	15,14	20	5:1
2	CuI	110	2	15	55	>99:1
3	Cp*RuCl(COD)	50	24	14	0	-
4	Cp*RuCl(COD)	110	24	14	0	-
5	Cp*RuCl(PPh_3_)_2_	80	8	14	33	<1:99
6	[Cp*RuCl]_4_	80	4	14	48	<1:99
7	[Cp*RuCl]_4_	110	4	14	41	<1:99

^a^ Reaction conditions: 0.3 mmol scale, 5 mol % of catalyst, toluene, 0.02 M; ^b^ Isolated yield.

**Scheme 2:**
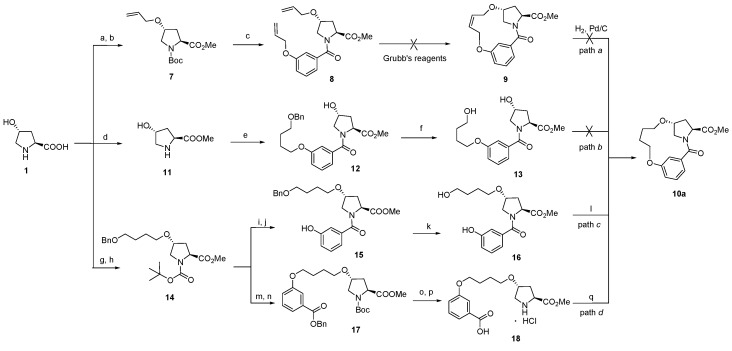
Macrocyclization of **10a** in four ways. *Reagents and conditions*: (a) (i) SOCl_2_, MeOH, reflux, overnight; (ii) (Boc)_2_O, Et_3_N, MeOH, 0 °C–r.t., 2 h, 96%; (b) 3-bromoprop-1-ene, NaH, DMF, 0 °C–r.t., 4 h, 35%; (c) (i) 3M HCl/EtOAc, r.t., 2 h; (ii) 3-(allyloxy)benzoic acid, HOBt, EDCI, Et_3_N, CH_2_Cl_2_, 0 °C–r.t., 2 h, 71%; (d) SOCl_2_, MeOH, reflux, overnight, 96%; (e) 3-(4-(benzyloxy)butoxy)benzoic acid, HOBt, EDCI, Et_3_N, CH_2_Cl_2_, 0 °C–r.t., 2 h, 75%; (f) H_2_, Pd/C, MeOH, r.t., 5 h, 92%; (g) (Boc)_2_O, Et_3_N, MeOH, 0 °C–r.t., 2 h, 95%; (h) (i) BnO(CH_2_)_4_Br, KI, NaH, DMF, 0 °C–r.t., overnight; (ii) CH_3_I, 50 °C, 2 h, 35%; (i) 3M HCl/EtOAc, r.t., 2 h, 94%; (j) 3-hydroxybenzoic acid, HOBt, EDCI, Et_3_N, CH_2_Cl_2_, 0 °C–r.t., 2 h, 73%; (k) H2, Pd/C, MeOH, r.t., 5 h, 92%; (l) ADDP, TBP, CH_2_Cl_2_, r.t., 4 h, 23%; (m) H_2_, Pd/C, MeOH, r.t., overnight, 90%; (n) benzyl 3-hydroxybenzoate, ADDP, TBP, CH_2_Cl_2_, 65%; (o) H_2_, Pd/C, MeOH, r.t., 5 h, 95%; (p) 3M HCl/EtOAc, r.t., 2 h, 93%; (q) HOBt, EDCI, Et_3_N, CH_2_Cl_2_, 0 °C–r.t., 1 h, 39%.

Encouraged by the results of macrocyclizations with azide-alkyne cycloadditions, we continued our investigation by performing the transformation of *trans*-4-hydroxy-l-proline (**1**) to **10a**. Retro-synthetic analysis indicates that 13-member macrocycle **10a** could be cyclized in four ways by ring-closing metathesis (RCM) [48,49,50,51,52]/hydrogenation reaction (path *a*), intramolecular Mitsunobu reaction (path *b,c*) and by amide formation reaction (path *d*). As shown in Scheme 2. *trans*-4-Hydroxy-l-proline (**1**) was sequentially esterified, *N*-protected with Boc group, alkylated with 3-bromopropene, *N*-deprotected and condensed under amide formation conditions to afford dialkene **8**. However, the cyclization of **8** failed under various conditions with both first and second generation Grubbs’ catalyst (path *a*). The results indicated that the conformation of **8** was not favored for the macrocyclization under RCM conditions. This is presumably caused by the rigid *meta* benzene junction that results in lower probability of encounter and increasing greater strain in the ansa-bridged macrocycles, which greatly reduces the effective morality (EM) of the terminal dienes [53,54,55].

With the RCM results and the conformational characteristics of the macrocycles, we turned to explore the possibility of macrocyclization using the Mitsunobu protocol. Unfortunately, the macrocyclization of path *b* failed under various Mitsunobu conditions (Table 2, Entries 1–3). This is due to the fact the pKa values of the protons of both hydroxyl group are bigger than the pKa value of the betaine intermediate during the reaction. On the other hand, the intramolecular Mitsunobu reaction of path *c* works smoothly and the desired 13-member macrocycle **10a** was obtained in a low yield of 7% using a mixture of triphenylphosphine and diethyl azodicarboxylate (DEAD) (Entry 4). Nevertheless, *N*,*N*,*N*′,*N*′-tetramethylazodicarboxamide (TMAD) was shown to enhance the reactivity of this nucleophile of pKa in inactivated systems, leading to higher overall yields (Entry 5). A combination of 1,1′-(azodicarbonyl)dipiperidine (ADDP) with tributyl phosphine (TBP) under argon atmosphere afforded a little better result with a 23% yield. (Entry 6). However, the desired macrocycle **10a** was difficult to isolate from the phosphine oxide formed in the cyclization and was still obtained in low yield. This could also be ascribed to the rigid junction between *meta*-benzenes and hydroxyproline which limits the rotational freedom of the molecular framework.

**Table 2:** Results of macrocyclization by Mitsunobu reactions and amide formation ^a^.

Entry	Path	Reagents	Time (h)	Yield (%) ^b^
1	b	DEAD, PPh_3_	24	0
2	b	TMAD, TBP	24	0
3	b	ADDP, TBP	24	0
4	c	DEAD, PPh_3_	4	10
5	c	TMAD, TBP	4	21
6	c	ADDP, TBP	4	23
7	d	HOBt, EDCI, Et_3_N	1	39
8	d	HATU, DIEA	1	39

^a^ Reactions were run on 0.3 mmol scale; ^b^ Isolated yield.

Excitingly, cyclization of **18** in path *d* works well with much higher yield and shorter time under standard amide formation conditions (Entry 7). No significant yield increase was found by using more effective HOAt-based reagent (Entry 8). The results indicate that the softer polymethylene linker between the rigid *meta*-benzene and hydroxyproline greatly reduces the strain in the ansa-bridged macrocycles and enhance the activity of head-to-tail cyclization. The effective morality of the terminal reactive groups is increased. The structure of **10a** was confirmed by ^1^H-NMR, ^13^C-NMR and HRMS.

**Scheme 3:**
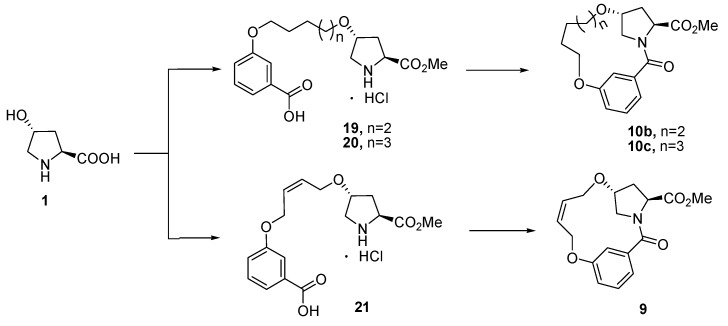
Synthesis of **8**, **9b** and **9c** under amide formation conditions.

We next turned our attention to the reactivity scope of macrocycle **10**. Under amide formation conditions, the formation of 14- and 15-member macrocycles **10b** and **10c** was achieved in moderate yields of 44% and 56%, respectively. The macrocyclization of **10** promoted us to reinvestigate the synthesis of **9** using the same macrocyclization strategy. Using *trans*-4-hydroxy-l-proline and *cis*-2-butene-1,4-diol as starting materials, macrocycle **9** was successfully obtained after five steps in 25% yield (Scheme 3).

The results indicate that the macrocyclization is not influenced by the configuration of the linear chain. Ring size is found to be an important factor that governs the yield of a head-to-tail macrocyclization. The yields of macrocycles increase with the increase of the rings in the range of 13–15 members because the strain energy of cyclization of 13-member macrocycles is much higher than that of 14- and 15-member rings, and the EM value of cyclization of 13-member macrocycles is much lower than that of 14- and 15-member rings.

### 2.2. In Vitro Anti-Tumor Screening

A small library derived from **6a**–**b**, **9** and **10a**–**c** was obtained by hydrolysis of the macrocycles and condensation with amines. The anti-tumor activities for lung cancer cell line A549, breast cancer cell line MDA-MB-231 and hepatocarcinoma cell line Hep G2 of all these new compounds were screened *in vitro* by an MTT assay. The IC_50_ values of the compounds are summarized in Table 3. Most of the compounds show some inhibitory activities against A549, MDA-MB-231 and Hep G2. Compounds **22**–**29** where the linker contains a triazole group showed moderate anti-tumor activity with IC_50_ values of 26.79–48.27 μmol/L. Compounds **30**–**33** containing alkene chains have antiproliferative effects on all three human tumor cell lines with IC_50_ values near 10 μmol/L, in particular compound **33** which showed the best activity against A549 cells with an IC_50_ value of 2.55 μmol/L, much better than the reference drug cisplatin with an IC_50_ value of 15.42 μmol/L. However, compounds **34**–**45** containing alkyl chains showed weaker anti-tumor activity than compounds **30**–**33**. In the future, further structure–activity relationship studies will be performed to determine how the substituents affected the anti-tumor activity and to design the best chemical structure in the future.

**Table 3:** Anti-tumor activities of macrocycles.

Compound		R	IC_50_ (μmol/L)		
			A549	MDA-MB-231	Hep G2
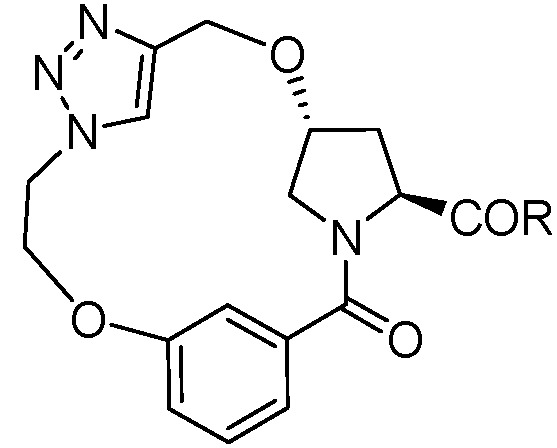	**22**	OH	32.24	45.14	44.17
	**23**	NH_2_	26.79	29.94	37.28
	**24**	NHPh	45.73	35.58	46.26
	**25**	NHCH_2_CH(CH_3_)_2_	37.73	42.47	48.27
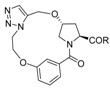	**26**	OH	37.11	34.28	44.88
	**27**	NH_2_	41.28	27.99	34.71
	**28**	NHPh	46.29	43.68	36.28
	**29**	NHCH_2_CH(CH_3_)_2_	32.18	43.19	37.31
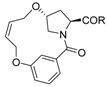	**30**	OH	11.23	10.26	23.21
	**31**	NH_2_	5.21	12.33	31.94
	**32**	NHPh	4.76	9.67	26.26
	**33**	NHCH_2_CH(CH_3_)_2_	2.55	11.87	41.10
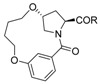	**34**	OH	>100	96.15	96.16
	**35**	NH_2_	89.54	90.54	91.47
	**36**	NHPh	54.31	85.32	95.39
	**37**	NHCH_2_CH(CH_3_)_2_	77.93	86.27	73.06
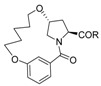	**38**	OH	>100	92.34	>100
	**39**	NH_2_	63.74	57.33	>100
	**40**	NHPh	87.99	76.36	68.39
	**41**	NHCH_2_CH(CH_3_)_2_	34.91	47.28	>100
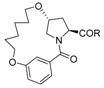	**42**	OH	>100	>100	87.39
	**43**	NH_2_	69.74	65.96	64.88
	**44**	NHPh	98.24	23.11	88.13
	**45**	NHCH_2_CH(CH_3_)_2_	93.57	88.69	89.58
Cisplatin			15.42	19.68	17.35

## 3. Materials and Methods

### 3.1. General Information

Pentamethylcyclopentadienylbis(triphenylphosphine)ruthenium(II) chloride [Cp*RuCl(PPh_3_)_2_], diethyl azodicarboxylate (DEAD), tri-*n*-butylphosphine (TBP), *N*,*N*,*N*′,*N*′-tetramethylazo-dicarboxamide (TMAD) and 1,1-(azodicarbonyl)-dipiperidine (ADDP), *N*-[(dimethylamino)-*H*-1,2,3-triazolo[4,5-b]pyridin-1-yl-methylene]-*N*-methylmethanaminium hexafluorophosphate (HATU) were purchased from Aldrich Chemical Company (Shanghai, China). Toluene was dried overnight over calcium chloride, filtered and distilled from sodium/benzophenone ketyl and degassed by three evacuation/refill cycles under Ar before use. Unless stated otherwise, other reagents and solvents were all purchased from commercial suppliers and were used without further purification. All reactions were monitored by TLC. Chromatography refers to open column chromatography (200–300 mesh). Melting points were recorded on a RY-1 microscopic melting apparatus (Tianjin, China) and are uncorrected. ^1^H-NMR and ^13^C-NMR spectra were recorded on Bruker 500 MHz and 125 Hz instruments (Bruker, Rheinstetten, Germany). Chemical shifts were reported in parts per million *δ* relative to tetramethylsilane. Mass spectra were performed on an Ultima Global spectrometer (Waters Corporation, Worcester, MA, USA) equipped with an ESI source.

### 3.2. Synthesis

#### 3.2.1. Procedure for the Preparation of Methyl (12*R*,14*S*)-16-Oxo-2,11-dioxa-5,6,7,15-tetraaza-tetracyclo[15.3.1.1^12,15^.0^5,9^]docosane-6,8,1(21),17,19-pentaene-14-carboxylate (**6a**)

A solution of compound **5** (0.11 g, 0.3 mmol) and copper(I) iodide (2.1 mg, 0.015 mmol) in anhydrous toluene (15 mL) was stirred at reflux under argon atmosphere for 2 hours. The solvent was removed under reduced pressure and the residue was subjected to column chromatography on silica gel (100–200 mesh) using petroleum/ethyl acetate as eluent to afford 1,4-disubstituted 1,2,3-triazole **6a** (0.061 g, 55%). The physical and spectral data for compound **6a** are listed below. ^1^H-NMR and ^13^C-NMR spectra are provided in the Supplementary Materials.White solid, m.p. 186–189 °C; ^1^H-NMR (-CDCl_3_): *δ* 7.74 (s, 1H), 7.25 (t, 1H, *J* = 7.9 Hz), 6.98 (dd, 1H, *J*_1_ = 2.2 Hz, *J_2_* = 8.3 Hz), 6.91 (d, 1H, *J* = 7.4 Hz), 6.21 (s, 1H), 4.95 (d, 1H, *J* = 14.1 Hz), 4.83–4.77 (m, 3H), 4.57–4.53 (m, 1H), 4.49–4.44 (m, 2H), 4.23–4.22 (m, 1H), 3.78 (s, 3H), 3.24–3.17 (m, 2H), 2.53–2.48 (m, 1H), 2.14–2.10 (m, 1H); ^13^C-NMR (-CDCl_3_): *δ* 172.7, 169.9, 158.8, 145.5, 137.5, 130.5, 124.2, 119.8, 119.3, 111.4, 80.2, 68.3, 64.5, 56.7, 55.0, 52.3, 51.8, 37.5; HRMS (ESI-TOF^+^): *m*/*z* Calcd. for C_18_H_21_N_4_O_5_ [M + H]^+^: 373.1512. Found: 373.1514.

#### 3.2.2. Procedure for the Preparation of Methyl (11*R*,13*S*)-15-Oxo-2,10-dioxa-5,6,7,14-tetraaza-tetracyclo[14.3.1.1^5,8^.1^11,14^]docosane-6,8(21),1(20),16,18-pentaene-13-carboxylate (**6b**)

A solution of compound **5** (0.11 g, 0.3 mmol) and [Cp*RuCl]_4_ (0.016 g, 0.015 mmol) in anhydrous toluene (15 mL) was stirred at 80 °C under argon atmosphere for 4 hours. The solvent was removed under reduced pressure and the resulting residue was purified by column chromatography with petroleum/ethyl acetate as eluting solvent to afford **6b** (0.054 g, 48%). The physical and spectral data for compound **6b** are listed below. ^1^H-NMR and ^13^C-NMR spectra are provided in the Supplementary Materials. White solid, m.p. 203–205 °C; ^1^H-NMR (CDCl_3_): *δ* 7.74 (s, 1H), 7.42 (t, 1H, *J* = 7.9 Hz), 7.22 (d, 1H, *J* = 7.5 Hz), 7.07 (dd, 1H, *J*_1_ = 2.3 Hz, *J*_2_ = 8.7 Hz), 7.00 (s, 1H), 5.10–5.04 (m, 1H), 4.94–4.91 (m, 1H), 4.74 (d, 1H, *J* = 11.5 Hz), 4.62–4.55 (m, 2H), 4.42 (d, 1H, *J* = 11.5 Hz), 4.36–4.31 (m, 1H), 4.22–4.21 (m, 1H), 4.04 (d, 1H, *J* = 13.6 Hz), 3.82 (s, 3H), 3.52 (dd, 1H, *J*_1_ = 2.9 Hz, *J*_2_ = 12.7 Hz), 2.64–2.59 (m, 1H), 2.41–2.36 (m, 1H). ^13^C-NMR (CDCl_3_): *δ* 172.5, 169.9, 137.9, 134.1, 132.9, 131.3, 120.3, 119.6, 109.9, 79.0, 64.6, 59.9, 56.4, 54.4, 53.4, 52.5, 43.4, 36.6. HRMS (ESI-TOF^+^): *m*/*z* Calcd. for C_18_H_21_N_4_O_5_ [M + H]^+^: 373.1512. Found: 373.1521.

#### 3.2.3. General Procedure for the Preparation of Methyl (4*Z*,8*R*,10*S*)-12-Oxo-2,7-dioxa-11-aza-tricyclo [11.3.1.1^8,11^]octadecane-4,1(17),13,15-tetraene-10-carboxylate (**9**) and Compounds **10a**–**c**

To a solution of **18**–**21** (0.3 mmol) in dichloromethane (15 mL), HOBt (0.36 mmol) was added slowly followed by EDCI (0.36 mmol) at 0 °C After stirring at r.t. for half an hour, a solution of triethylamine (0.75 mmol) in dichloromethane (2 mL) was added dropwise at 0 °C. Then the mixture was stirred at r.t. for half an hour. After adding 10 mL of water, the mixture was extracted with dichloromethane (3 × 5 mL). The combined organic extracts were dried over anhydrous sodium sulfate and concentrated *in vacuo*. The residue was subjected to column chromatography on silica gel (100–200 mesh) using petroleum/ethyl acetate as eluent to afford **9** and **10a**–**c**, with yields ranging from 25% to 56%. The physical and spectral data for compounds **9** and **10a**–**c** are listed below. ^1^H-NMR and ^13^C-NMR spectra are provided in the Supplementary Materials.

**9**: Yellow oil, yield 25%, ^1^H-NMR (CDCl_3_): *δ* 7.23 (t, 1H, *J* = 7.9 Hz), 7.07 (d, 1H, *J* = 7.4 Hz), 6.94 (s,1H), 6.84 (d, 1H, *J* = 8.0 Hz), 5.76–5.72 (m, 1H), 5.68–5.66 (m, 1H), 4.74 (s, 1H, *J* = 8.2 Hz), 4.39 (d, 2H, *J* = 6.0 Hz), 4.07–4.03 (m, 1H), 3.99–3.90 (m, 2H), 3.71 (s, 3H), 3.64–3.52 (m, 2H), 2.42–2.38 (m, 1H), 2.06–2.02 (m, 1H). ^13^C-NMR (CDCl_3_): *δ* 172.7, 169.6, 158.2, 140.0, 129.5, 128.0, 119.9, 116.9, 113.3, 64.8, 63.8, 57.7, 54.3, 52.4, 35.2.; HRMS (ESI-TOF^+^): *m*/*z* Calcd. for C_17_H_20_NO_5_ [M + H]^+^: 318.1341. Found: 318.1343.

*Methyl (8R,10S)-12-oxo-2,7-dioxa-11-aza-tricyclo[11.3.1.1^8,11^]octadecane-1(17),13,15-triene-10-carboxylate* (**10a**): White solid, m.p. 142–144 °C, yield 39%, ^1^H-NMR (CDCl_3_): *δ* 7.26 (t, 1H, *J* = 8.2 Hz), 7.09 (d, 1H, *J* = 7.4 Hz), 7.01 (s, 1H), 6.84 (dd, 1H, *J*_1_ = 2.3 Hz, *J*_2_ = 8.3 Hz), 4.85 (t, 1H, *J* = 8.5 Hz), 4.01 (br, 1H), 3.87–3.82 (m, 2H), 3.77 (s, 3H), 3.64 (s, 2H), 3.54–3.48 (m, 1H), 3.26–3.24 (m, 1H), 2.48–2.44 (m, 1H), 2.11–2.05 (m, 1H), 1.76–1.67 (m, 2H), 1.64–1.62 (m, 2H). ^13^C-NMR (CDCl_3_) *δ* 172.8, 170.0, 158.7, 137.0, 129.2, 119.4, 117.0, 112.6, 68.4, 67.5, 57.6, 54.8, 52.3, 35.3, 26.3, 25.8.; HRMS (ESI-TOF^+^): *m*/*z* Calcd. for C_17_H_22_NO_5_ [M + H]^+^: 320.1498. Found: 320.1505.

*Methyl (9R,11S)-13-oxo-2,8-dioxa-12-aza-tricyclo[12.3.1.1^9,12^]nonadecane-1(18),14,16-triene-11-carboxylate* (**10b**): Yellow oil, yield 44%, ^1^H-NMR (CDCl_3_): *δ* 7.18 (t, 1H, *J* = 7.8 Hz), 7.01 (d, 1H, *J* = 7.6 Hz), 6.98 (s, 1H), 6.85 (d, 1H, *J* = 8.0 Hz) , 4.68 (t, 1H, *J* = 8.1 Hz), 3.97 (br, 1H), 3.89–3.85 (m, 1H), 3.83–3.80 (m, 1H), 3.69 (s, 3H), 3.65 (dd, 1H, *J*_1_ = 4.0 Hz, *J*_2_ = 11.5 Hz), 3.47 (d, 1H, *J* = 11.3 Hz), 3.38–3.36 (m, 1H), 3.19–3.14 (m, 1H), 2.37 (t, 1H, *J* = 10.5 Hz), 2.03–1.97 (m, 1H), 1.68–1.64 (m, 2H), 1.49–1.44 (m, 2H), 1.42–1.38 (m, 2H). ^13^C-NMR (CDCl_3_): δ 172.6, 169.9, 158.9, 136.8, 129.1, 119.3, 117.5, 112.0, 68.7, 67.7, 57.7, 54.9, 52.2, 34.6, 29.3, 28.8, 22.8.; HRMS (ESI-TOF^+^): *m*/*z* Calcd. for C_18_H_24_NO_5_ [M + H]^+^: 334.1654. Found: 334.1653.

*Methyl (10R,12S)-14-oxo-2,9-dioxa-13-aza-tricyclo[13.3.1.1^10,13^]eicosane-1(19),15,17-triene-12-carboxylate* (**10c**) Yellow oil, yield 54%, ^1^H-NMR (CDCl_3_): *δ* 7.21 (t, 1H, *J* = 7.8 Hz), 7.03 (d, 1H, *J* = 7.6 Hz), 7.00 (s, 1H), 6.85 (dd, 1H, *J*_1_ = 2.0 Hz, *J*_2_ = 8.3 Hz), 4.73 (t, 1H, *J* = 8.2 Hz), 3.96 (br, 1H), 3.87–3.83 (m, 2H), 3.71 (s, 3H), 3.63–3.60 (m, 1H), 3,54-3.51 (m, 1H), 3.38–3.35 (m, 1H), 3.16–3.12 (m, 1H), 2.36 (t, 1H, *J* = 10.0 Hz), 2.04–1.98 (m, 1H), 1.67–1.63 (m, 2H), 1.46–1.41 (m, 3H), 1.34–1.30 (m, 3H). ^13^C-NMR (CDCl_3_): *δ* 172.7, 169.9, 158.9, 136.9, 129.2, 119.3, 116.8, 112.9, 68.8, 67.8, 57.7, 54.7, 52.3, 35.1, 29.5, 29.0, 25.8, 25.6.; HRMS (ESI-TOF^+^): *m*/*z* Calcd. for C_19_H_26_NO_5_ [M + H]^+^: 348.1811. Found: 348.1813.

#### 3.2.4. General Procedure for the Preparation of Compounds **22**–**45**

To a suspension of macrocyclic ester **6a**–**b**, **9** or **10a**–**c** (1 mmol) in methanol (2 mL), a solution of NaOH (4.8 mg, 1.2 mmol) in water (0.5 mL) was added slowly. After stirring at r.t. for 3 h, the mixture was evaporated to remove solvent and acidified with 3N HCl to pH = 3, the precipitated product **22**, **26**, **30**, **34**, **38** or **42** was isolated by filtration and dried under reduced pressure.

To a solution of macrocyclic acid **22**, **26**, **30**, **34**, **38** or **42** (0.1 mmol) in dichloromethane (0.5 mL), HOBt (0.15mmol) was added slowly followed by EDCI (0.15 mmol) at 0 °C After stirring at r.t. for half an hour, a solution of amine (0.12 mmol) and triethylamine (0.25 mmol) in dichloromethane (0.2 mL) was added dropwise at 0 °C. Then the mixture was stirred at r.t. for 1 h. After adding 1 mL of water, the mixture was extracted with dichloromethane (3 × 0.3 mL). The combined organic extracts were dried over anhydrous sodium sulfate and concentrated *in vacuo*. The residue was subjected to column chromatography on silica gel (100–200 mesh) using petroleum/ethyl acetate as eluent to afford macrocyclic amide **23**–**25**, **27**–**29**, **31**–**33**, **35**–**37**, **39**–**41** or **43**–**45**. The physical and spectral data for compounds **22**–**45** are listed below. ^1^H-NMR spectra are provided in the Supplementary Materials.

*(11R,13S)-15-Oxo-2,10-dioxa-5,6,7,14-tetraaza-tetracyclo[14.3.1.1^5,8^.1^11,14^]docosane-6,8(21),1(20),16,18-pentaene-13-carboxylic acid* (**22**): White solid, m.p. 210–212 °C, yield 91%, ^1^H-NMR (DMSO-*d_6_*): *δ* 8.24 (s, 1H), 7.23 (t, 1H, *J* = 7.8 Hz), 6.93 (d, 1H, *J* = 7.4 Hz), 6.64 (d, 1H, *J* = 7.2 Hz), 6.07 (s, 1H), 4.89–4.86 (m, 1H), 4.84–4.82 (m, 1H), 4.65 (s, 2H), 4.38–4.20 (m, 4H), 3.07–3.04 (m, 1H), 2.95–2.93 (m, 1H), 2.35–2.31 (m, 1H), 2.03–1.98 (m, 1H). HRMS (ESI-TOF^+^): *m*/*z* Calcd. for C_17_H_19_N_4_O_5_ [M + H]^+^: 359.1355. Found: 359.1364.

*(11R,13S)-15-Oxo-2,10-dioxa-5,6,7,14-tetraaza-tetracyclo[14.3.1.1^5,8^.1^11,14^]docosane-6,8(21),1(20),16,18-pentaene-13-carboxamide* (**23**): White solid, m.p. 257–259 °C, yield 75%, ^1^H-NMR (DMSO-*d_6_*): *δ* 8.18 (s, 1H), 7.40–6.81 (m, 4H), 6.09 (s, 1H), 6.64 (d, 1H, *J* = 7.2 Hz), 5.75 (s, 1H), 4.91–4.90 (m, 1H), 4.82–4.79 (m, 1H), 4.64 (s, 2H), 4.41–4.15 (m, 4H), 3.14–3.12 (m, 1H), 2.96–2.94 (m, 1H), 2.25–2.24 (m, 1H), 1.98–1.94 (m, 1H). HRMS (ESI-TOF^+^): *m*/*z* Calcd. for C_17_H_20_N_5_O_4_ [M + H]^+^: 358.1515. Found: 358.1512.

*(11R,13S)-15-Oxo-N-phenyl-2,10-dioxa-5,6,7,14-tetraaza-tetracyclo[14.3.1.1^5,8^.1^11,14^]docosane-6,8(21),1(20),16,18-pentaene-13-carboxamide* (**24**): White solid, m.p. 258–260 °C, yield 82%, ^1^H-NMR (CDCl_3_): *δ* 9.57 (s, 1H), 7.40–6.81 (m, 4H), 7.59 (s, 1H), 7.55–7.53 (m, 2H), 7.31–7.21 (m, 3H), 7.08 (t, 1H, *J* = 7.5 Hz), 7.02 (d, 1H, *J* = 7.9 Hz), 6.76 (d, 1H, *J* = 7.3 Hz), 6.31 (s, 1H), 5.00 (t, 1H, *J* = 7.3 Hz), 4.91 (d, 1H, *J* = 14.0 Hz), 4.81–4.78 (m, 2H), 4.57–4.55 (m, 2H), 4.38 (d, 1H, *J* = 14.1 Hz), 4.25–4.24 (m, 1H), 3.37–3.21 (m, 2H), 2.93–2.91 (m, 1H), 2.27–2.22 (m, 1H). HRMS (ESI-TOF^+^): *m*/*z* Calcd. for C_23_H_24_N_5_O_4_ [M + H]^+^: 434.1828. Found: 434.1822.

*(11R,13S)-15-Oxo-N-isobutyl-2,10-dioxa-5,6,7,14-tetraaza-tetracyclo[14.3.1.1^5,8^.1^11,14^]docosane-6,8(21),1(20),16,18-pentaene-13-carboxamide* (**25**): White solid, m.p. 270–272 °C, yield 79%, ^1^H-NMR (CDCl_3_): *δ* 7.56 (s, 1H), 7.31 (s, 1H), 7.23–7.22 (m, 1H), 7.01 (d, 1H, *J* = 7.6 Hz), 6.74 (d, 1H, *J* = 7.2 Hz), 6.29 (s, 1H), 4.90–4.79 (m, 4H), 4.55–4.54 (m, 2H), 4.36 (d, 1H, *J* = 14.1 Hz), 4.20 (s, 1H), 3.31–3.28 (m, 1H), 3.20–3.15 (m, 2H), 3.04–3.01 (m, 1H), 2.85–2.80 (m, 1H), 2.20–2.16 (m, 1H), 1.79–1,76 (m, 1H), 0.89 (d, 6H, *J* = 6.3 Hz). HRMS (ESI-TOF^+^): *m*/*z* Calcd. for C_21_H_28_N_5_O_4_ [M + H]^+^: 414.2141. Found: 414.2135.

*(12R,14S)-16-Oxo-2,11-dioxa-5,6,7,15-tetraaza-tetracyclo[15.3.1.1^12,15^.0^5,9^]docosane-6,8,1(21),17,19-pentaene-14-carboxylic acid* (**26**): White solid, m.p. 222–225 °C, yield 90%, ^1^H-NMR (DMSO-*d_6_*): *δ* 7.83 (s, 1H), 7.44 (t, 1H, *J* = 7.8 Hz), 7.09 (d, 1H, *J* = 8.2 Hz), 7.04 (d, 1H, *J* = 7.3 Hz), 6.82 (s, 1H), 4.89–4.83 (m, 1H), 4.73–4.65 (m, 3H), 4.56–4.49 (m, 2H), 4.36–4.32 (m, 1H), 4.26–4.25 (m, 1H), 4.02–4.00 (m, 1H), 3.28–3.26 (m, 1H), 2.47–2.42 (m, 1H), 2.30–2.26 (m, 1H). HRMS (ESI-TOF^+^): *m*/*z* Calcd. for C_17_H_19_N_4_O_5_ [M + H]^+^: 359.1355. Found: 359.1346.

*(12R,14S)-16-Oxo-2,11-dioxa-5,6,7,15-tetraaza-tetracyclo[15.3.1.1^12,15^.0^5,9^]docosane-6,8,1(21),17,19-pentaene-14-carboxamide* (**27**): White solid, m.p. 258–260 °C, yield 77%, ^1^H-NMR (DMSO-*d_6_*): *δ* 7.82 (s, 1H), 7.50 (s, 1H), 7.44 (t, 1H, *J* = 7.8 Hz), 7.12–7.08 (s, 3H), 6.76 (s, 1H), 4.85–4.83 (m, 1H), 4.70–4.58 (m, 3H), 4.52–4.51 (m, 2H), 4.38–4.37 (m, 1H), 4.21 (s, 1H), 3.92–3.90 (m, 1H), 3.29–3.26 (m, 1H), 2.34–2.25 (m, 2H). HRMS (ESI-TOF^+^): *m*/*z* Calcd. for C_17_H_20_N_5_O_4_ [M + H]^+^: 358.1515. Found: 358.1516.

*(12R,14S)-16-Oxo-N-phenyl-2,11-dioxa-5,6,7,15-tetraaza-tetracyclo[15.3.1.1^12,15^.0^5,9^]docosane-6,8,1(21),17,19-pentaene-14-carboxamide* (**28**): White solid, m.p. 229–232 °C, yield 80%, ^1^H-NMR (CDCl_3_): *δ* 9.95 (s, 1H), 7.72 (s, 1H), 7.62–7.60 (m, 2H), 7.44–7.33 (m, 3H), 7.17 (d, 1H, *J* = 7.4 Hz), 7.14–7.08 (m, 3H), 5.10–5.07 (m, 1H), 5.04–5.01 (m, 1H), 4.80 (d, 1H, *J* = 11.6 Hz), 4.72–4.66 (m, 1H), 4.61–4.59 (m, 1H), 4.48 (d, 1H, *J* = 11.6 Hz), 4.31–4.26 (m, 2H), 4.04–4.01 (d, 1H), 3.29–3.23 (m, 2H), 2.33–2.28 (m, 1H). HRMS (ESI-TOF^+^): *m*/*z* Calcd. for C_23_H_24_N_5_O_4_ [M + H]^+^: 434.1828. Found: 434.1833.

*(12R,14S)-16-Oxo-N-isobutyl-2,11-dioxa-5,6,7,15-tetraaza-tetracyclo[15.3.1.1^12,15^.0^5,9^]docosane-6,8,1(21),17,19-pentaene-14-carboxamide* (**29**): White solid, m.p. 200–202 °C, yield 68%, ^1^H-NMR (CDCl_3_): *δ* 7.70 (s, 1H), 7.67 (s, 1H), 7.41(t, 1H, *J* = 7.8 Hz), 7.11 (d, 1H, *J* = 7.3 Hz), 7.07–7.06 (m, 2H), 5.03–4.99 (m, 1H), 4.92–4.90 (m, 1H), 4.77 (d, 1H, *J* = 11.6 Hz), 4.68–4.66 (m, 1H), 4.60–4.56 (m, 1H), 4.44 (d, 1H, *J* = 11.6 Hz), 4.32–4.28 (m, 1H), 4.23–4.22 (m, 1H), 3.96–3.94 (m, 1H), 3.25–3.22 (m, 1H), 3.21–3.07 (m, 3H), 2.25–2.21 (m, 1H), 1.85–1.79 (m, 1H). HRMS (ESI-TOF^+^): *m*/*z* Calcd. for C_21_H_28_N_5_O_4_ [M + H]^+^: 414.2141. Found: 414.2147.

*(4Z,8R,10S)-12-Oxo-2,7-dioxa-11-aza-tricyclo[11.3.1.1^8,11^]octadecane-4,1(17),13,15-tetraene-10-carboxylic acid* (**30**): White solid, m.p. 176–178 °C, yield 93%, ^1^H-NMR (CDCl_3_): *δ* 7.15 (t, 1H, *J* = 7.7 Hz), 6.67 (d, 1H, *J* = 7.2 Hz), 6.84 (s, 1H), 6.77 (d, 1H, *J* = 7.0 Hz), 5.63–5.59 (m, 2H), 4.62 (t, 1H, *J* = 7.9 Hz), 4.33–4.32 (m, 2H), 4.00 (s, 1H), 3.87–3.86 (m, 2H), 3.55–3.54 (m, 1H), 3.45–3.42 (m, 1H), 2.33–2.31 (m, 1H), 2.03–2.02 (m, 1H). HRMS (ESI-TOF^+^): *m*/*z* Calcd. for C_16_H_18_NO_5_ [M + H]^+^: 304.1185. Found: 304.1176.

*(4Z,8R,10S)-12-Oxo-2,7-dioxa-11-aza-tricyclo[11.3.1.1^8,11^]octadecane-4,1(17),13,15-tetraene-10-carboxamide* (**31**): Colorless oil, yield 84%, ^1^H-NMR (CDCl_3_): *δ* 7.31 (d, 1H, *J* = 7.3 Hz), 7.15 (d, 1H, *J* = 7.5 Hz), 7.02 (s, 1H), 6.94 (t, 1H, *J* = 8.3 Hz), 5.74–5.73 (m, 2H), 5.49 (s, 1H), 4.93 (t, 1H, *J* = 8.2 Hz), 4.63–4.55 (m, 2H), 4.05–3.97 (m, 2H), 3.92–3.89 (m, 1H), 3.69 (s, 1H), 2.63–2.59 (m, 1H), 2.36–2.31 (m, 1H). HRMS (ESI-TOF^+^): *m*/*z* Calcd. for C_16_H_19_N_2_O_4_ [M + H]^+^: 303.1345. Found: 303.1340.

*(4Z,8R,10S)-12-Oxo-N-phenyl-2,7-dioxa-11-aza-tricyclo[11.3.1.1^8,11^]octadecane-4,1(17),13,15-tetraene-10-carboxamide* (**32**): White solid, m.p. 190–194 °C, yield 77%, ^1^H-NMR (CDCl_3_): *δ* 9.90 (s, 1H), 7.36–7.31 (m, 3H), 7.18 (d, 1H, *J* = 7.4 Hz), 7.00–6.98 (m, 1H), 6.95 (s, 1H), 6.91–6.88 (m, 2H), 6.73 (t, 1H, *J* = 7.3 Hz), 5.73–5.64 (m, 2H), 5.16–5.12 (m, 1H), 5.02–4.98 (m, 1H), 4.63–4.60 (m, 1H), 4.43–4.39 (m, 1H), 4.14–4.13 (m, 1H), 3.88–3.85 (m, 1H), 3.75–3.73 (m, 1H), 3.55 (d, 1H, *J* = 11.7 Hz), 2.72–2.68 (m, 1H), 2.31–2.26 (m, 1H). HRMS (ESI-TOF^+^): *m*/*z* Calcd. for C_22_H_23_N_2_O_4_ [M + H]^+^: 379.1658. Found: 379.1662.

*(4Z,8R,10S)-12-Oxo-N-isobutyl-2,7-dioxa-11-aza-tricyclo[11.3.1.1^8,11^]octadecane-4,1(17),13,15-tetraene-10-carboxamide* (**33**): White solid, m.p. 201–204 °C, yield 71%, ^1^H-NMR (DMSO-*d_6_*): *δ* 7.96 (s, 1H), 7.33 (t, 1H, *J* = 7.9Hz), 7.10 (d, 1H, *J* = 7.6 Hz), 6.99 (s, 1H), 6.95 (d, 1H, *J* = 8.3 Hz), 5.70–5.66 (m, 2H), 4.54 (t, 1H, *J* = 8.5 Hz), 4.78–4.47 (m, 2H), 4.07 (s, 1H), 4.01–3.94 (m, 2H), 3.67–3.65 (m, 1H), 3.49 (d, 1H, *J* = 11.8 Hz), 2,98–2.94 (m, 1H), 2.87–2.85 (m, 1H), 2.28–2.27 (m, 1H), 1.71–1.68 (m, 1H), 0.82 (d, 6H, *J* = 6.5 Hz). HRMS (ESI-TOF^+^): *m*/*z* Calcd. for C_20_H_27_N_2_O_4_ [M + H]^+^: 359.1971. Found: 359.1977.

*(8R,10S)-12-Oxo-2,7-dioxa-11-aza-tricyclo[11.3.1.1^8,11^]octadecane-1(17),13,15-triene-10-carboxylic acid* (**34**): White solid, m.p. 187–190 °C, yield 90%, ^1^H-NMR (DMSO-*d*_6_): δ 7.33 (t, 1H, *J* = 7.8 Hz), 7.02 (d, 1H, *J* = 7.6 Hz), 6.99 (s, 1H), 6.94 (d, 1H, *J* = 8.2 Hz), 4.52 (t, 1H, *J* = 8.8 Hz), 4.00 (s, 1H), 3.85–3.83 (m, 2H), 3.58–3.56 (m, 1H), 3.51–3.46 (m, 2H), 3.17–3.14 (m, 1H), 2.37–2.33 (m, 1H), 2.03–1.98 (m, 1H), 1.66–1.64 (m, 1H), 1.50–1.43 (m, 3H). HRMS (ESI-TOF^+^): *m*/*z* Calcd. for C_16_H_20_NO_5_ [M + H]^+^: 306.1341. Found: 306.1344.

*(8R,10S)-12-Oxo-2,7-dioxa-11-aza-tricyclo[11.3.1.1^8,11^]octadecane-1(17),13,15-triene-10-carboxamide* (**35**): White solid, m.p. 60–63 °C, yield 69%, ^1^H-NMR (DMSO-*d_6_*): *δ* 7.48 (s, 1H), 7.33 (t, 1H, *J* = 7.4 Hz), 7.15 (d, 1H, *J* = 7.0 Hz), 7.03 (d, 1H, *J* = 6.3 H), 6.94 (d, 1H, *J* = 8.1 Hz), 4.49 (t, 1H, *J* = 8.5 Hz), 3.95 (s, 1H), 3.84–3.79 (m, 2H), 3.60–3.58 (m, 1H), 3.47–3.45 (m, 2H), 3.16–3.12 (m, 1H), 2.30–2.25 (m, 1H), 1.94–1.90 (m, 1H), 1.67–1.64 (m, 1H), 1.48–1.43 (m, 3H). HRMS (ESI-TOF^+^): *m*/*z* Calcd. for C_16_H_21_N_2_O_4_ [M + H]^+^: 305.1501. Found: 305.1508.

*(8R,10S)-12-Oxo-N-phenyl-2,7-dioxa-11-aza-tricyclo[11.3.1.1^8,11^]octadecane-1(17),13,15-triene-10-carboxamide* (**36**): White solid, m.p. 119–123 °C, yield 76%, ^1^H-NMR (CDCl_3_): *δ* 9.63 (s, 1H), 7.59 (m, 2H), 7.32–7.30 (m, 3H), 7.12–7.10 (m, 2H), 7.00 (d, 1H, *J* = 7.5 Hz), 6.93–6.91 (m, 1H), 5.15 (t, 1H, *J* = 7.7 Hz), 4.02 (s, 1H), 3.93–3.89 (m, 2H), 3.80–3.78 (m, 1H), 3.56–3.54 (m, 1H), 3.48–3.46 (m, 1H), 3.20–3.16 (m, 1H), 2.90–2.85 (m, 1H), 2.28–2.24 (m, 1H), 1.81–1.78 (m, 1H), 1.69–1,64 (m, 4H). HRMS (ESI-TOF^+^): *m*/*z* Calcd. for C_22_H_25_N_2_O_4_ [M + H]^+^: 381.1814. Found: 381.1811.

*(8R,10S)-12-Oxo-N-isobutyl-2,7-dioxa-11-aza-tricyclo[11.3.1.1^8,11^]octadecane-1(17),13,15-triene-10-carboxamide* (**37**): White solid, m.p. 207–210 °C, yield 79%, ^1^H-NMR (CDCl_3_): *δ* 7.36 (t, 1H, *J* = 5.6 Hz), 7.30–7.29 (m, 1H), 7.07 (s, 1H), 6.97 (d, 1H, *J* = 7.4 Hz), 6.90 (d, 1H, *J* = 7.8 Hz), 4.96 (t, 1H, *J* = 8.3 Hz), 3.97 (s, 1H), 3.88–3.85 (m, 2H), 3,72–3.69 (m, 1H), 3.54–3.52 (m, 1H), 3.44–3.43 (m, 1H), 3.16–3.08 (m, 3H), 3.79–3.94 (m, 1H), 2.22–2.18 (m, 1H), 1.83–1.79 (m, 1H), 1.69–1,61 (m, 4H), 0.92 (d, 6H, *J* = 6.4 Hz). HRMS (ESI-TOF^+^): *m*/*z* Calcd. for C_20_H_29_N_2_O_4_ [M + H]^+^: 361.2127. Found: 361.2130.

*(9R,11S)-13-Oxo-2,8-dioxa-12-aza-tricyclo[12.3.1.1^9,12^]nonadecane-1(18),14,16-triene-11-carboxylic acid* (**38**): White solid, m.p. 187–190 °C, yield 93%, ^1^H-NMR (CDCl_3_): *δ* 7.32 (t, 1H, *J* = 7.4 Hz), 7.10 (s, 1H), 7.06 (d, 1H, *J* = 6.5 Hz), 6.99 (d, 1H, *J* = 7.2 Hz), 4.90 (t, 1H, *J* = 7.4 Hz), 4.05-3.97 (m, 2H), 4.01–3.97 (m, 1H), 3.93–3.91 (m, 1H), 3.71–3.66 (m, 1H), 3.62–3.60 (m, 1H), 3.48–3.47 (m, 1H), 3.26–3.25 (m, 1H), 2.45–2.43 (m, 2H), 1.78–1.76 (m, 2H), 1.60–1.57 (m, 2H), 1.48–1.45 (m, 2H). HRMS (ESI-TOF^+^): *m*/*z* Calcd. for C_17_H_22_NO_5_ [M + H]^+^: 320.1498. Found: 320.1495.

*(9R,11S)-13-Oxo-2,8-dioxa-12-aza-tricyclo[12.3.1.1^9,12^]nonadecane-1(18),14,16-triene-11-carboxamide* (**39**): White solid, m.p. 53–55 °C, yield 68%, ^1^H-NMR (DMSO-*d*_6_): *δ* 7.45 (s, 1H), 7.36–7.32 (m, 1H), 7.35 (d, 1H, *J* = 8.9 Hz), 7.04–7.02 (m, 1H), 7.01 (s, 2H), 4.42 (t, 1H, *J* = 8.4 Hz), 4.02–4.01 (m, 1H), 3.97–3.96 (m, 1H), 3.92–3.91 (m, 1H), 3.61–3.59 (m, 1H), 3.46–3.44 (m, 2H), 3.17–3.16 (m, 1H), 2.38–2.36 (m, 1H), 2.01–1.95 (m, 1H), 1.45–1.44 (m, 4H), 1.38–1.34 (m, 2H). HRMS (ESI-TOF^+^): *m*/*z* Calcd. for C_17_H_23_N_2_O_4_ [M + H]^+^: 319.1658. Found: 319.1651.

*(9R,11S)-13-Oxo-N-phenyl-2,8-dioxa-12-aza-tricyclo[12.3.1.1^9,12^]nonadecane-1(18),14,16-triene-11-carboxamide* (**40**): White solid, m.p. 88–91 °C, yield 65%, ^1^H-NMR (CDCl_3_): *δ* 9.62 (s, 1H), 7.60-7.58 (m, 2H), 7.32–7.29 (m, 3H), 7.14 (s, 1H), 7.10 (d, 1H, *J* = 7.2 Hz), 7.02–6.98 (m, 2H), 5.11 (t, 1H, *J* = 8.0 Hz), 4.07–4.06 (m, 1H), 4.03–4.00 (m, 1H), 3.95–3.93 (m, 1H), 3.75–3.72 (m, 1H), 3.53–3.50 (m, 1H), 3.45–3.43 (m, 1H), 3.24–3.18 (m, 1H), 2.87–2.82 (m, 1H), 2.78–2.23 (m, 1H), 1.79–1.76 (m, 2H), 1.60–1.55 (m, 2H), 1.51–1.47 (m, 1H), 1.44–1.41 (m, 1H). HRMS (ESI-TOF^+^): *m*/*z* Calcd. for C_23_H_27_N_2_O_4_ [M + H]^+^: 395.1971. Found: 395.1972.

*(9R,11S)-13-Oxo-N-isobutyl-2,8-dioxa-12-aza-tricyclo[12.3.1.1^9,12^]nonadecane-1(18),14,16-triene-11-carboxamide* (**41**): White solid, m.p. 176–179 °C, yield 71%, ^1^H-NMR (CDCl_3_): *δ* 7.31–7.29 (m, 1H), 7.08 (s, 1H), 6.98–6.96 (m, 2H), 4.91 (t, 1H, *J* = 8.0 Hz), 4.02–3.97 (m, 2H), 3.93–3.89 (m, 1H), 3.66–3.64 (m, 1H), 3.51–3.49 (m, 1H), 3.42–3.39 (m, 1H), 3.16–3.08 (m, 3H), 2.73–2.69 (m, 1H), 2.21–2.17 (m, 1H), 1.82–1.80 (m, 1H), 1.78–1.74 (m, 2H), 1.57–1.53 (m, 2H), 1.48–1.37 (m, 2H), 0.92 (d, 6H, *J* = 6.7 Hz). HRMS (ESI-TOF^+^): *m*/*z* Calcd. for C_21_H_31_N_2_O_4_ [M + H]^+^: 375.2284. Found: 375.2287.

*(10R,12S)-14-Oxo-2,9-dioxa-13-aza-tricyclo[13.3.1.1^10,13^]eicosane-1(19),15,17-triene-12-carboxylic acid* (**42**): White solid, m.p. 201–203 °C, yield 91%, ^1^H-NMR (DMSO-*d_6_*): *δ* 7.36 (t, 1H, *J* = 7.9 Hz), 7.05–7.00 (m, 2H), 6.95 (s, 1H), 4.47 (t, 1H, *J* = 8.3 Hz), 4.00 (s, 1H), 3.91–3.89 (m, 2H), 3.60–3.47 (m, 3H), 3.15–3.14 (m, 1H), 2.36–2.32 (m, 1H), 1.99–1.98 (m, 1H), 1.62–1.61 (m, 1H), 1.42–1.38 (m, 2H), 1.30–1.15 (m, 4H). HRMS (ESI-TOF^+^): *m*/*z* Calcd. for C_18_H_24_NO_5_ [M + H^+^]: 334.1654. Found: 334.1658.

*(10R,12S)-14-Oxo-2,9-dioxa-13-aza-tricyclo[13.3.1.1^10,13^]eicosane-1(19),15,17-triene-12-carboxamide* (**43**): White solid, m.p. 51–53°C, yield 66%, ^1^H-NMR (DMSO-*d_6_*): *δ* 7.47 (s, 1H), 7.34 (t, 1H, *J* = 7.5 Hz), 7.14 (d, 1H, *J* = 6.0 Hz), 7.03–7.00 (m, 3H), 4.46 (t, 1H, *J* = 8.5 Hz), 4.03–4.00 (m, 2H), 3.95 (s, 1H), 3.90–3.88 (m, 2H), 3.61–3.45 (m, 3H), 3.08–3.07 (m, 1H), 2.27–2.23 (m, 1H), 1.93–1.91 (m, 1H), 1.62–1.61 (m, 2H), 1.37–1.36 (m, 2H), 1.28–1.27 (m, 2H), 1.18–1.15 (m, 2H). HRMS (ESI-TOF^+^): *m*/*z* Calcd. for C_18_H_25_N_2_O_4_ [M + H]^+^: 333.1814. Found: 333.1819.

*(10R,12S)-14-Oxo-N-phenyl-2,9-dioxa-13-aza-tricyclo[13.3.1.1^10,13^]eicosane-1(19),15,17-triene-12-carboxamide* (**44**): White solid, m.p. 102–104 °C, yield 69%, ^1^H-NMR (CDCl_3_): *δ* 9.66 (s, 1H), 7.59–7.57 (m, 2H), 7.33–7.28 (m, 3H), 7.11 (s, 1H), 7.08 (t, 1H, *J* = 7.4 Hz), 7.01–6.97 (m, 2H), 5.12 (t, 1H, *J* = 8.1 Hz), 4.02 (s, 1H), 3.99–3.94 (m, 2H), 3.76–3.74 (m, 1H), 3.47–3.44 (m, 2H), 3.17–3.13 (m, 1H), 2.88–2.83 (m, 1H), 2.27–2.22 (m, 1H), 1.79–1.77 (m, 2H), 1.52–1.51 (m, 2H), 1.42–1.37 (m, 2H), 1.29–1.26 (m, 2H). HRMS (ESI-TOF^+^): *m*/*z* Calcd. for C_24_H_29_N_2_O_4_ [M + H^+^]: 409.2127. Found: 409.2130.

*(10R,12S)-14-oxo-N-Isobutyl-2,9-dioxa-13-aza-tricyclo[13.3.1.1^10,13^]eicosane-1(19),15,17-triene-12-carboxamide* (**45**): White solid, m.p. 83–85 °C, yield 74%, ^1^H-NMR (CDCl_3_): *δ* 7.34 (t, 1H, *J* = 5.5 Hz), 7.32–7.28 (m, 1H), 7.07 (s, 1H), 6.97–6.95 (m, 2H), 4.93 (t, 1H, *J* = 8.1 Hz), 3.97–3.90 (m, 3H), 3.67 (m, 1H), 3.43–3.41 (m, 2H), 3.17–3.06 (m, 3H), 2.76–2.71 (m, 1H), 2.20–2.16 (m, 1H), 1.83–1.79 (m, 1H), 1.50–1.48 (m, 2H), 1.40–1.38 (m, 4H), 1.26–1.23 (m, 2H), 0.92 (d, 6H, *J* = 6.4 Hz). HRMS (ESI-TOF^+^): *m*/*z* Calcd. for C_22_H_33_N_2_O_4_ [M + H]^+^: 389.2440. Found: 389.2439.

### 3.3. Cytotoxicity Assays

The human lung cancer cell line A549, breast cancer cell line MDA-MB-231 and hepatocarcinoma cell line Hep G2 were cultured in Dulbecco’s modified Eagle’s medium (DMEM) supplemented with antibiotics (penicillin 50 U/mL; streptomycin 50 µg/mL) and 10% FCS. The incubation was at 37 °C in a humidified atmosphere of 5% CO_2_ in air before experiments. Cells were first seeded at a density of 8000 cells/well in a 96-well plate for 48 hours. Solutions containing respective concentrations of compounds were added into wells and incubation continued for another 24 hours. After that, the MTT (3-(4,5-dimethylthiazol-2-yl)-2,5-diphenyltetrazolium bromide) dye stock solution (10 μL, 5 mg/mL) was added to each well. After 4 h, the supernatant was removed and DMSO (100 μL) was added to solubilize the MTT. The absorbance was measured at a wave length of 490 nm (A_490nm_) on an ELISA microplate reader. Results were expressed as IC_50_ values.

## 4. Conclusions

In summary, we have established the cyclization of 13- to 15-member macrocycles which contain alkyl-alkyl ether and alkyl-aryl ether linkers based on incorporation of 1,3-(*meta*)-benzene rings into hydroxyproline under azide-alkyne cycloaddition and amide formation conditions. The macrocyclization strategy will be further used to expand the scope and diversity of these macrocyclic derivatives. The initial biological results provided preliminary basis for further structural optimization of hydroxyproline-based macrocycles as promising inhibitors against lung cancer cell line A549, breast cancer cell line MDA-MB-231 and hepatocarcinoma cell line Hep G2. Efforts to optimize the structure of compound **33** to further improve its potency are ongoing.

## Supplementary Materials

Supplementary materials are available online at www.mdpi.com/1420-3049/21/2/212/s1.
