# Supplementary material for: Synthetic Strategy and Anti-Tumor Activities of Macrocyclic Scaffolds Based on 4-Hydroxyproline
Source: Molecules. 2016 Feb 15;21(2):212. doi: 10.3390/molecules21020212 (PMC6274554; doi:10.3390/molecules21020212)
Supplement: Supplementary file 1 [file molecules-21-00212-s001.pdf]

Supplementary Materials: Synthetic Strategy and Anti-Tumor Activities of Macrocyclic Scaffolds Based on 4-Hydroxyproline

Guorui Cao, Kun Yang, Yue Li, Longjiang Huang and Dawei Teng

Table of Contents

1. General S2

2. Spectra of macrocycle **6a**–**b** S3–S4

3. Spectra of macrocycle **9** S5

4. Spectra of macrocycle **10a**–**c** S6–S8

5. Spectra of macrocycle **22**–**45** S9–S20

General

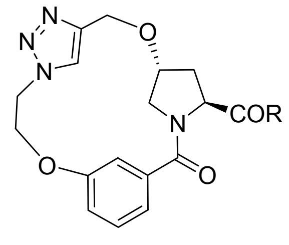

6a R = OCH_3
22 R = OH
23 R = NH_2
24 R = NHPh
25 R = $NHCH_2CH(CH_3)_2$

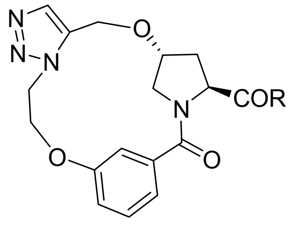

6b R = OCH_3
26 R = OH
27 R = NH_2
28 R = NHPh
29 R = $NHCH_2CH(CH_3)_2$

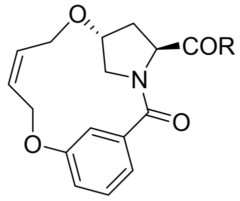

9 R = OCH_3
30 R = OH
31 R = NH_2
32 R = NHPh
33 R = $NHCH_2CH(CH_3)_2$

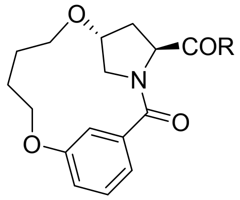

10a R = OCH_3
34 R = OH
35 R = NH_2
36 R = NHPh
37 R = $NHCH_2CH(CH_3)_2$

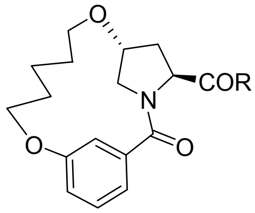

10b R = OCH_3
38 R = OH
39 R = NH_2
40 R = NHPh
41 R = $NHCH_2CH(CH_3)_2$

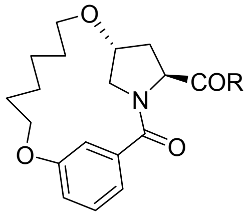

10c R = OCH_3
42 R = OH
43 R = NH_2
44 R = NHPh
45 R = $NHCH_2CH(CH_3)_2$

Figure S1. Target macrocycles.

Spectra of Macrocycle 6a–b

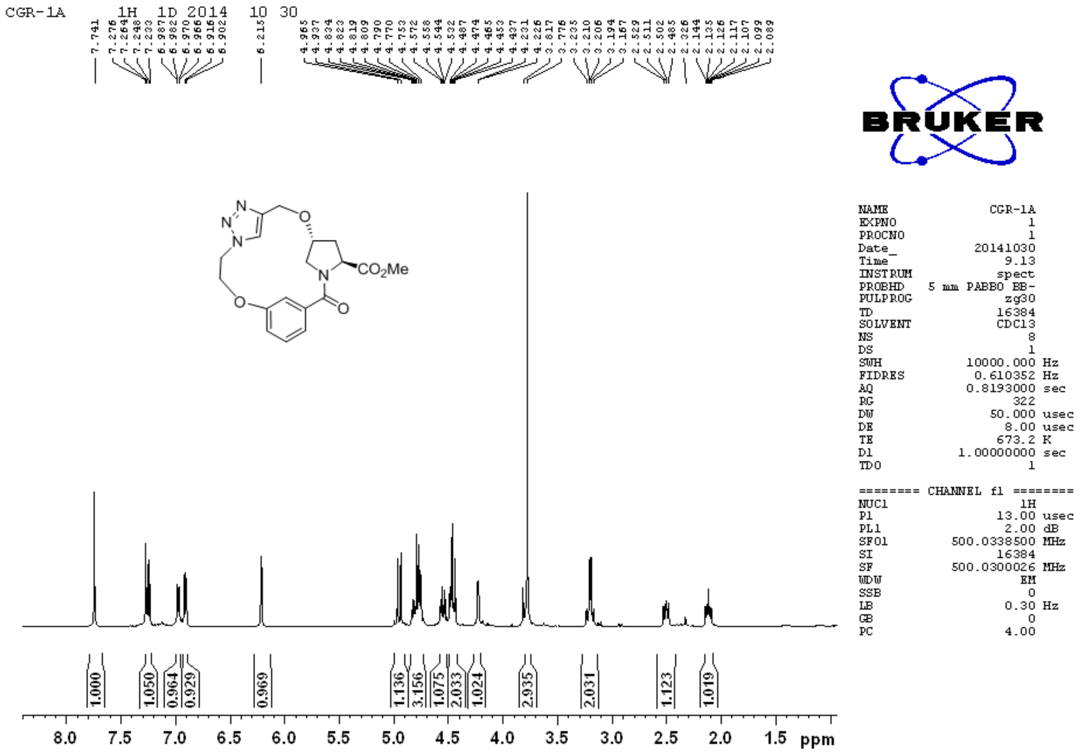

Figure S2. ^{1}H-NMR Spectrum of **6a**.

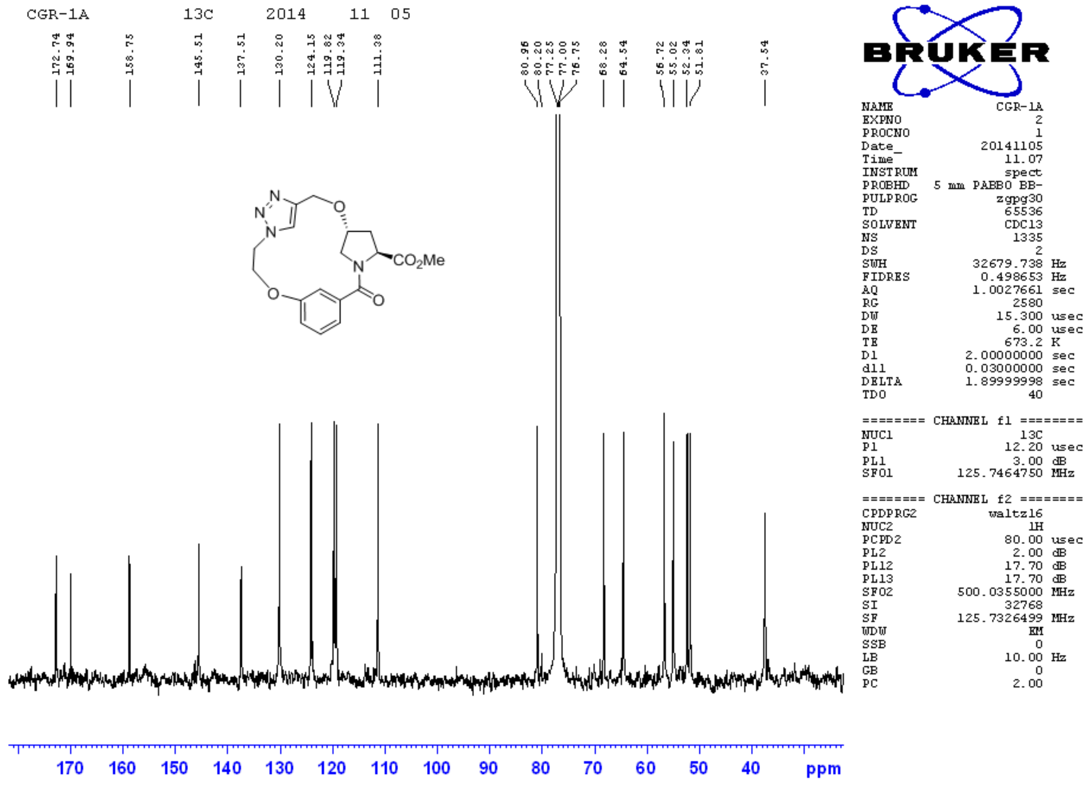

Figure S3. ^{13}C-NMR Spectrum of **6a**.

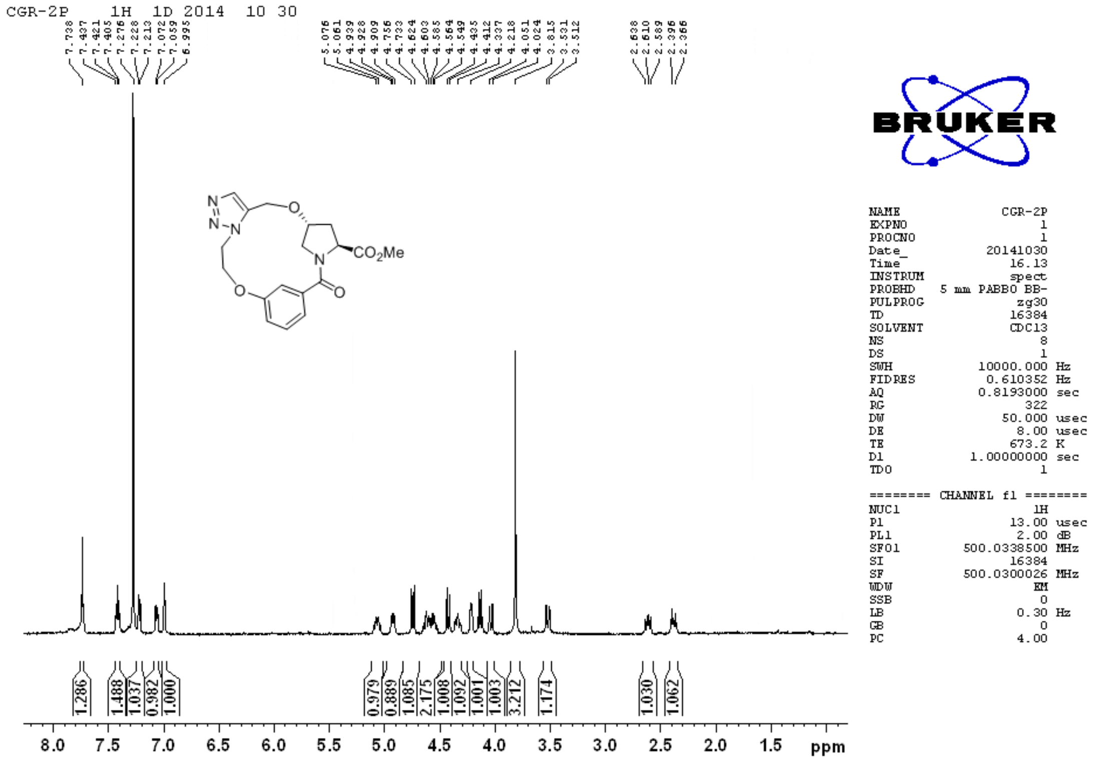

Figure S4. ^{1}H-NMR Spectrum of **6b**.

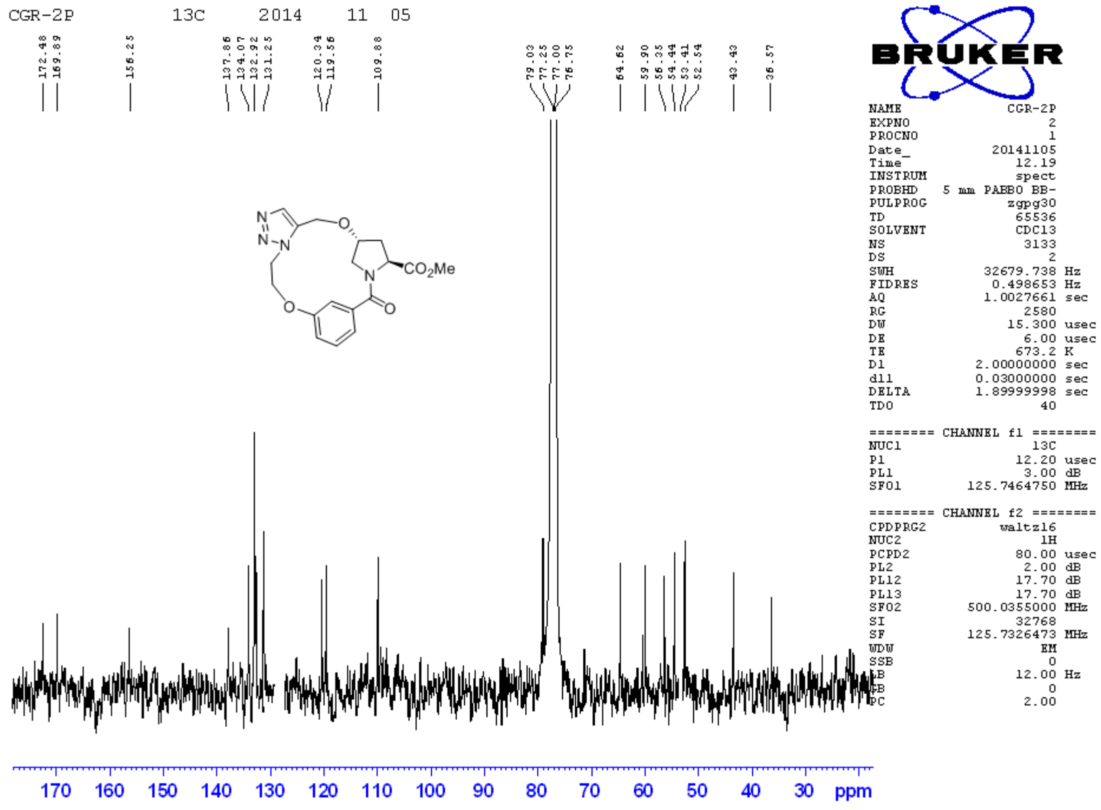

Figure S5. ^{13}C-NMR Spectrum of **6b**.

Spectra of macrocycle 9

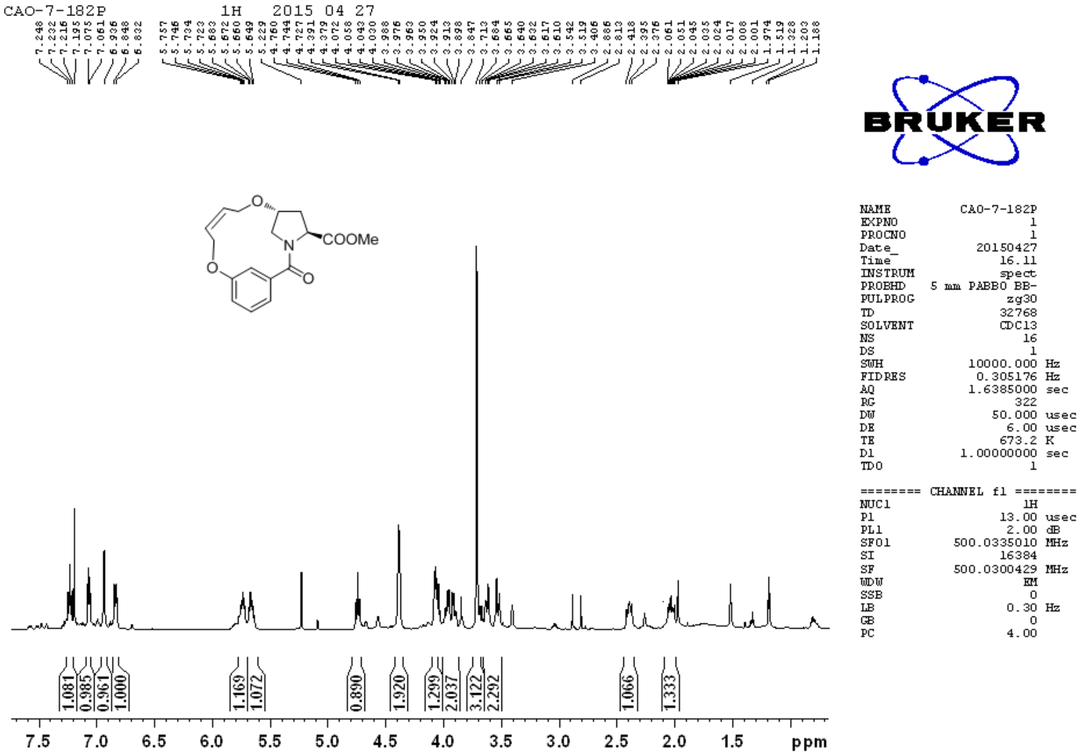

Figure S6. ^{1}H-NMR Spectrum of **9**.

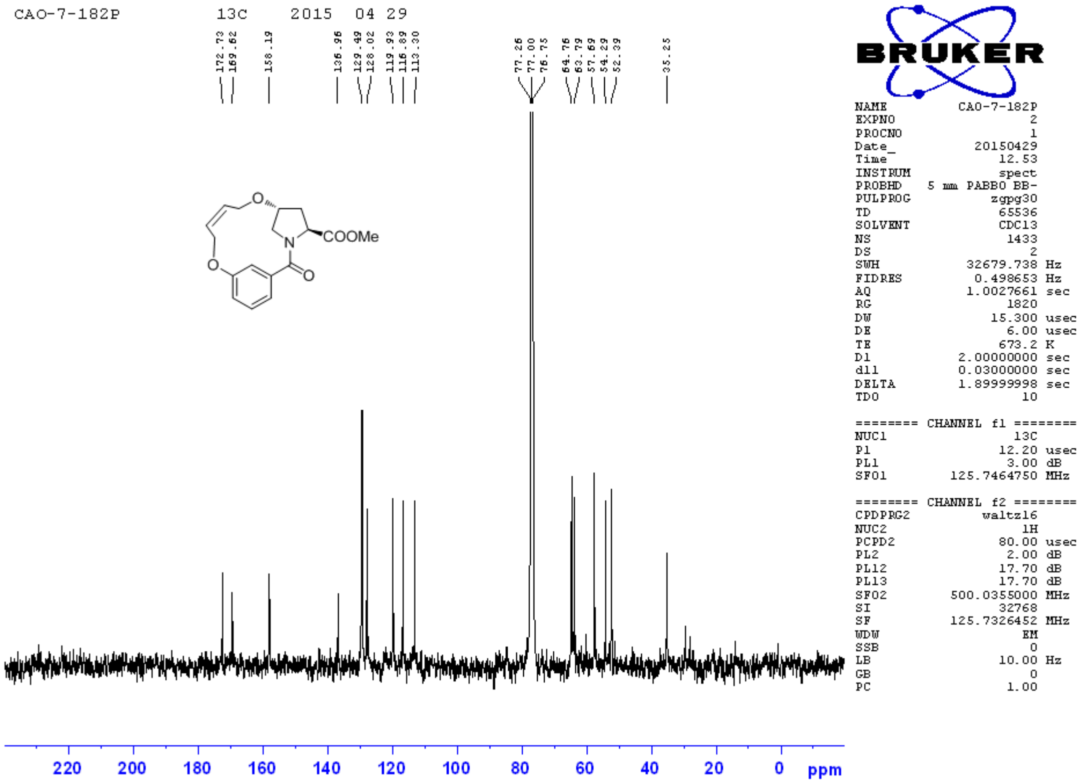

Figure S7. ^{13}C-NMR Spectrum of **9**.

Spectra of macrocycle 10a–c

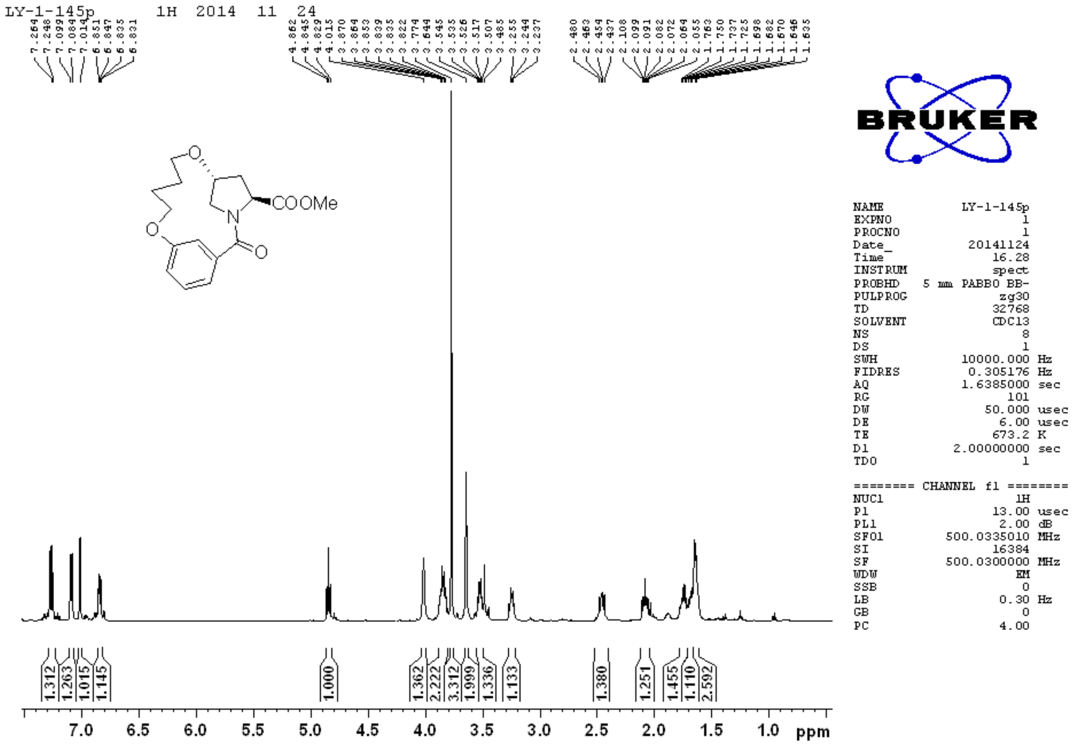

Figure S8. ^{1}H-NMR Spectrum of **10a**.

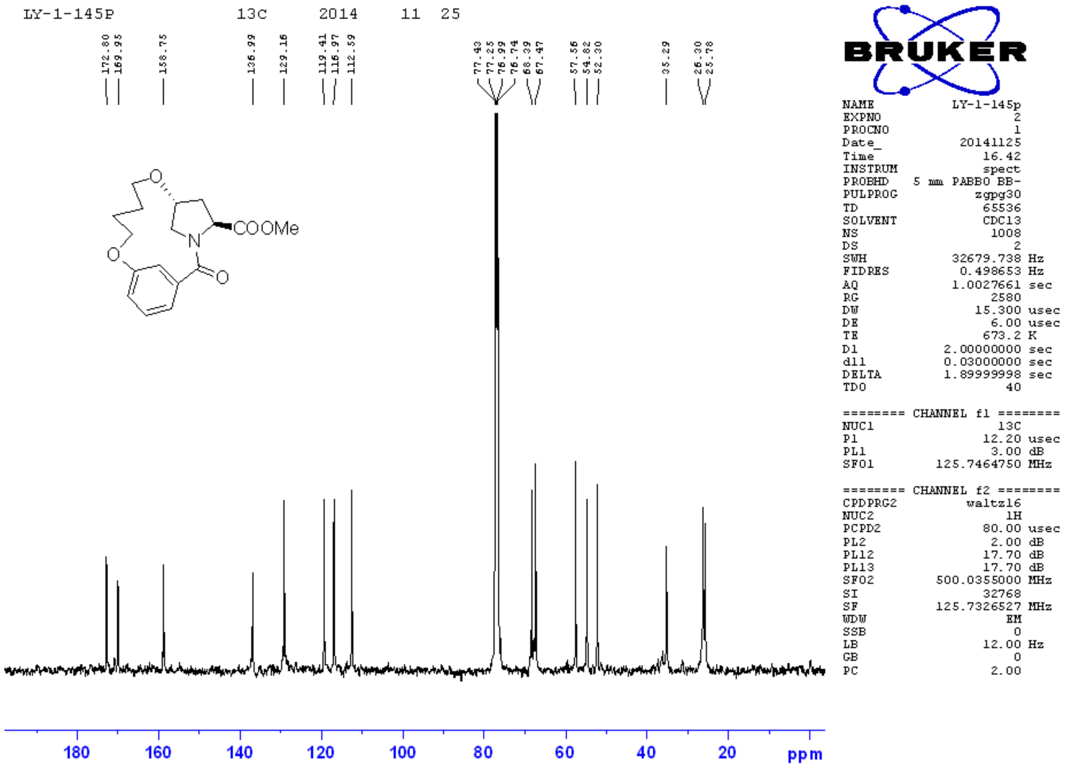

Figure S9. ^{13}C-NMR Spectrum of **10a**.

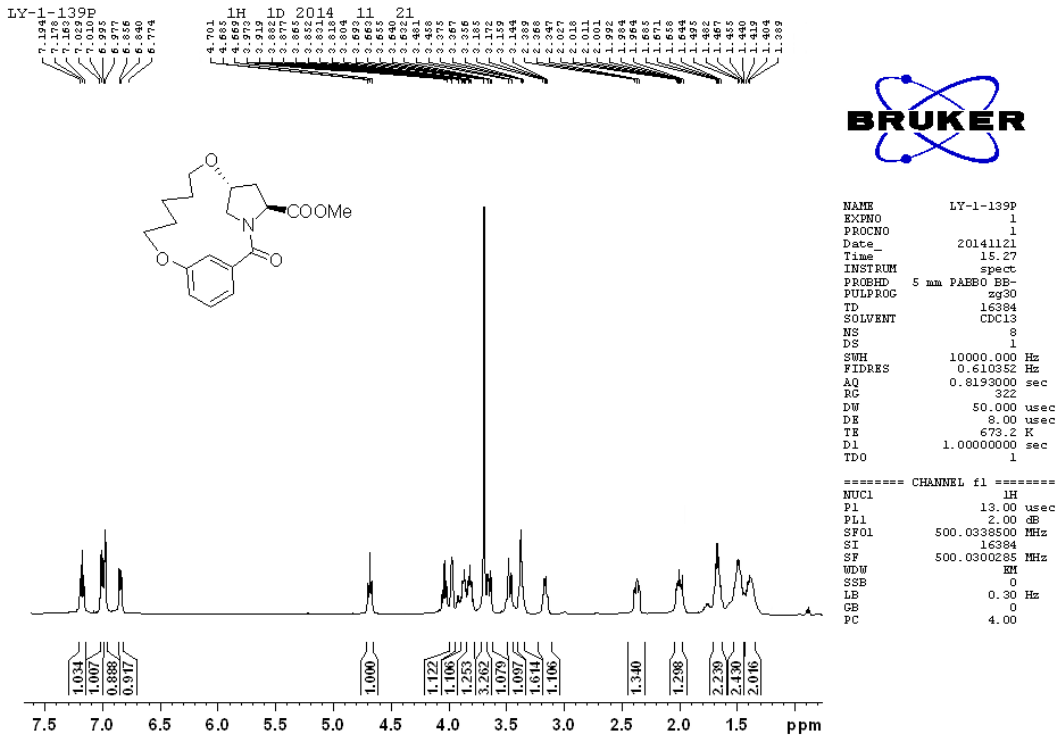

Figure S10. ^{1}H-NMR Spectrum of **10b**.

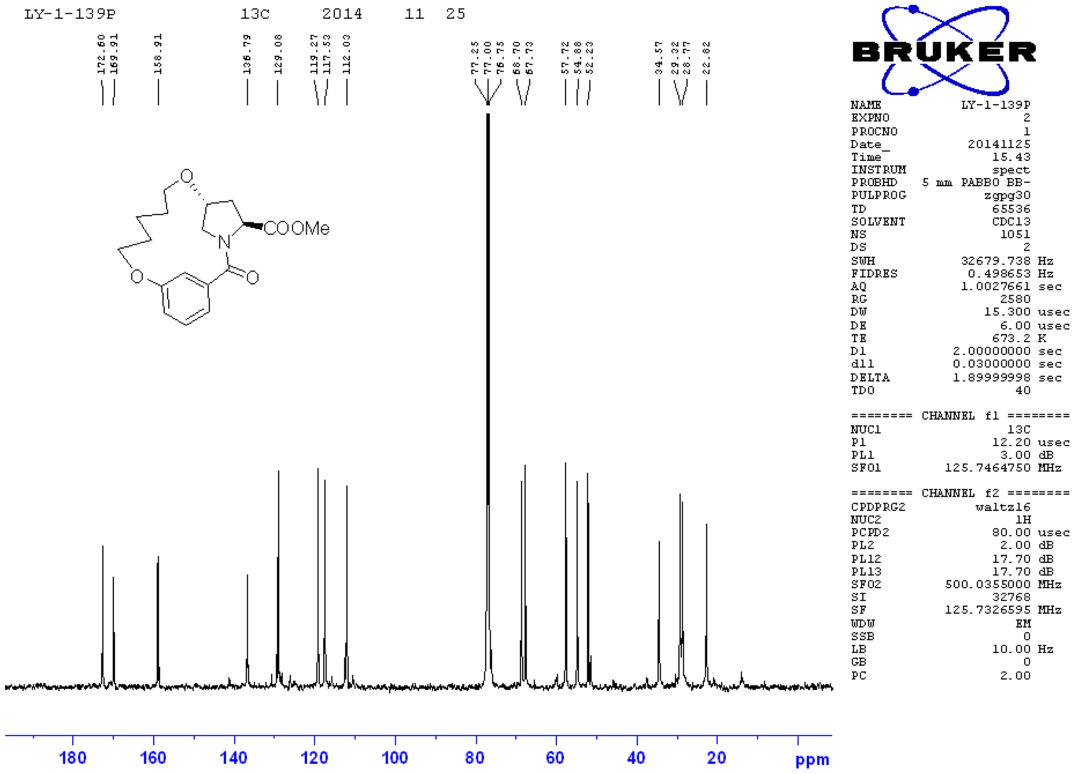

Figure S11. ^{13}C-NMR Spectrum of **10b**.

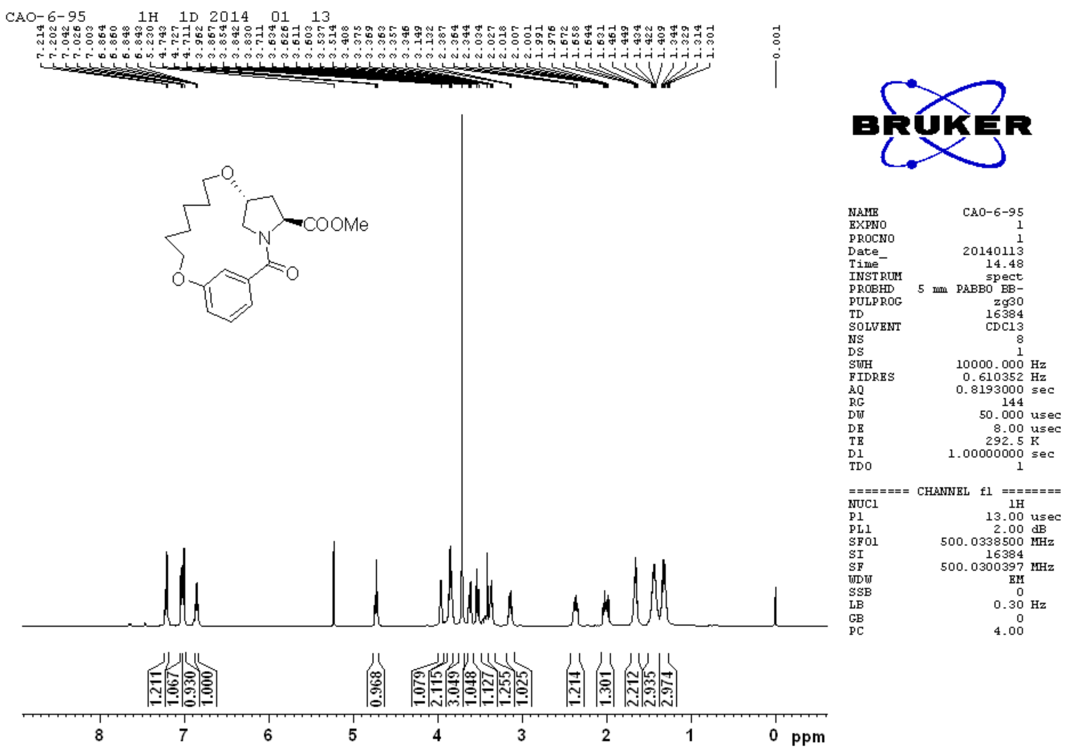

Figure S12. ^{1}H-NMR Spectrum of **10c**.

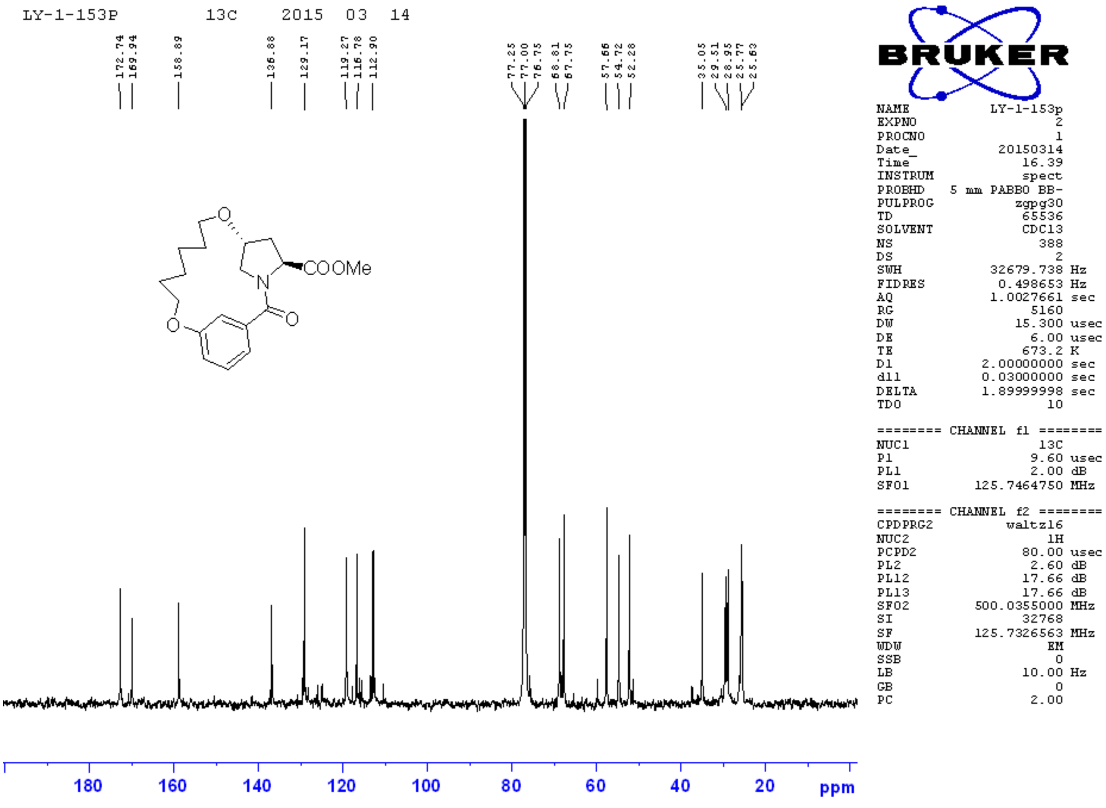

Figure S13. ^{13}C-NMR Spectrum of **10c**.

Spectra of macrocycle 22–45

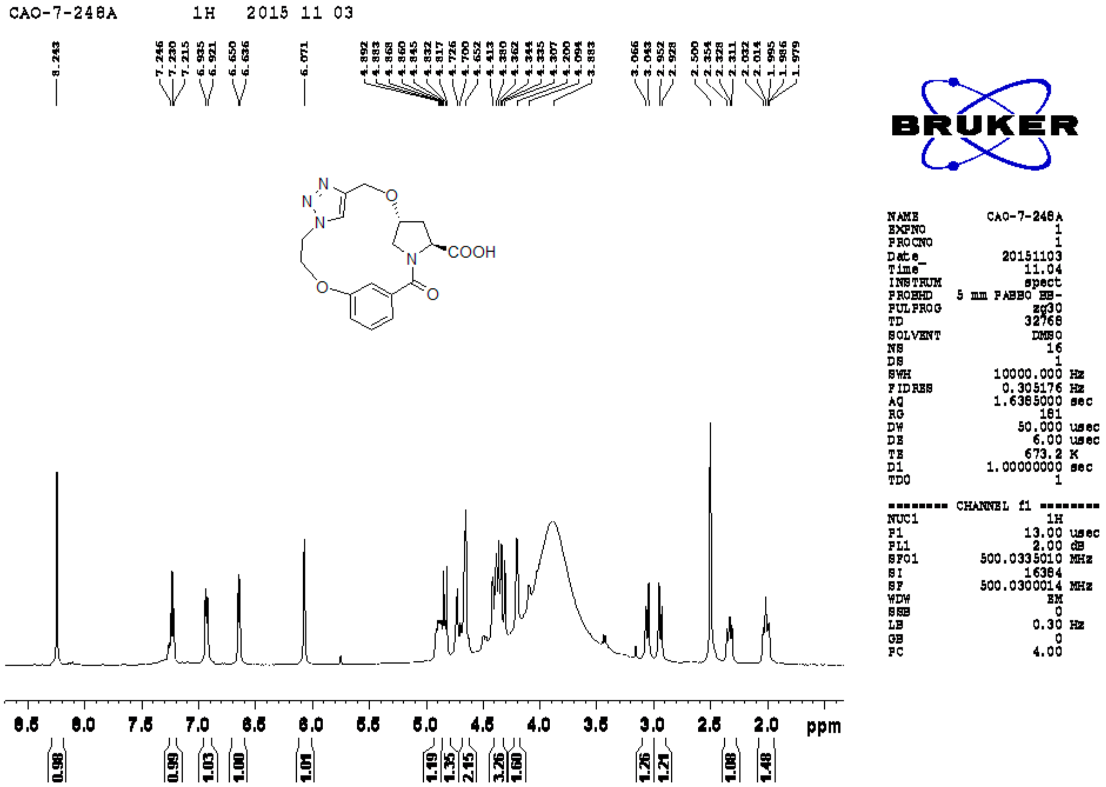

Figure S14. ^{1}H-NMR Spectrum of **22**.

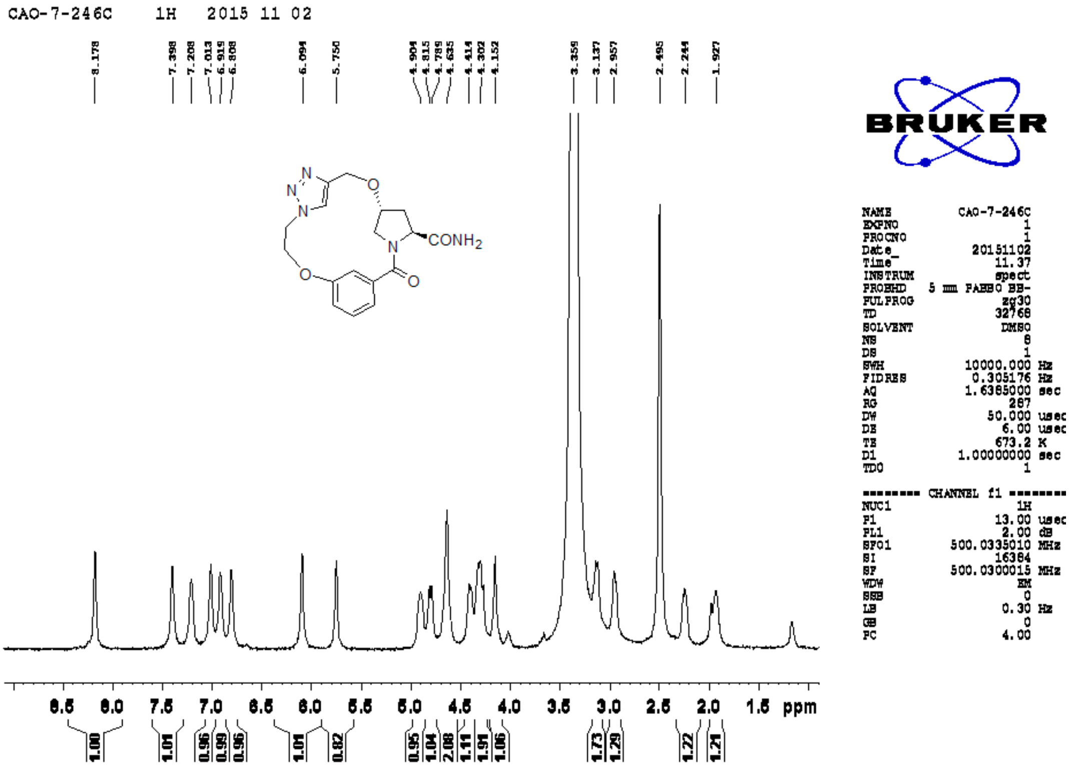

Figure S15. ^{1}H-NMR Spectrum of **23**.

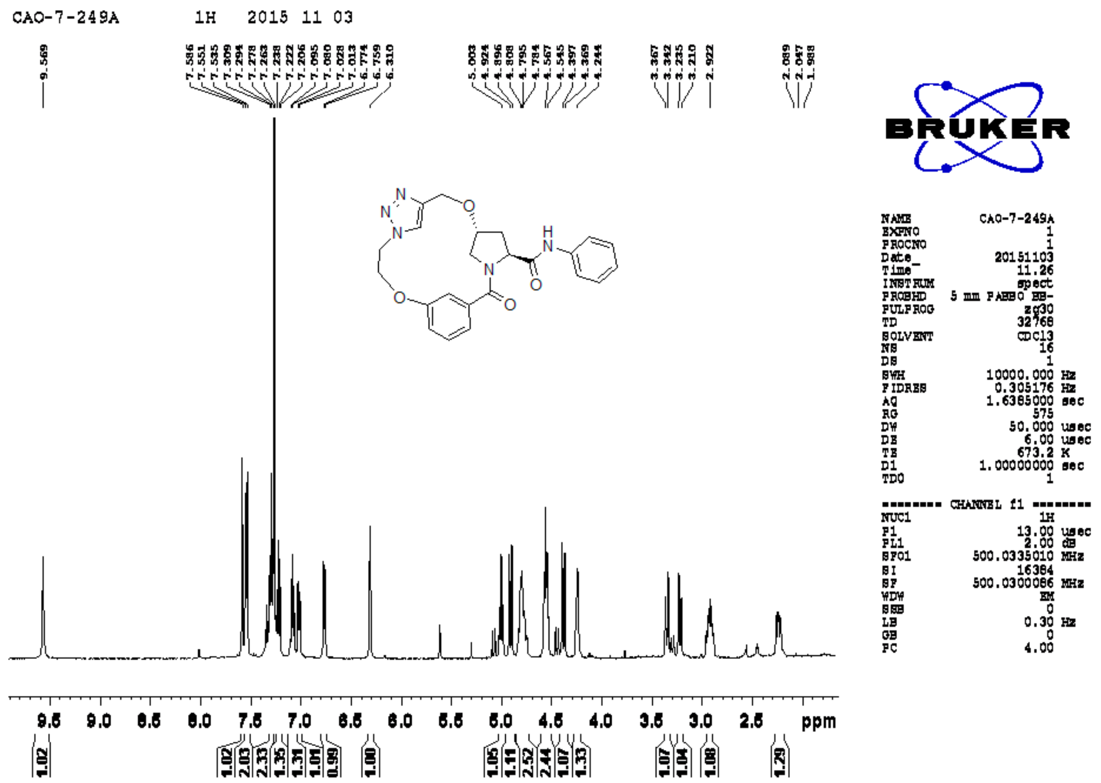

Figure S16. ^{1}H-NMR Spectrum of **24**.

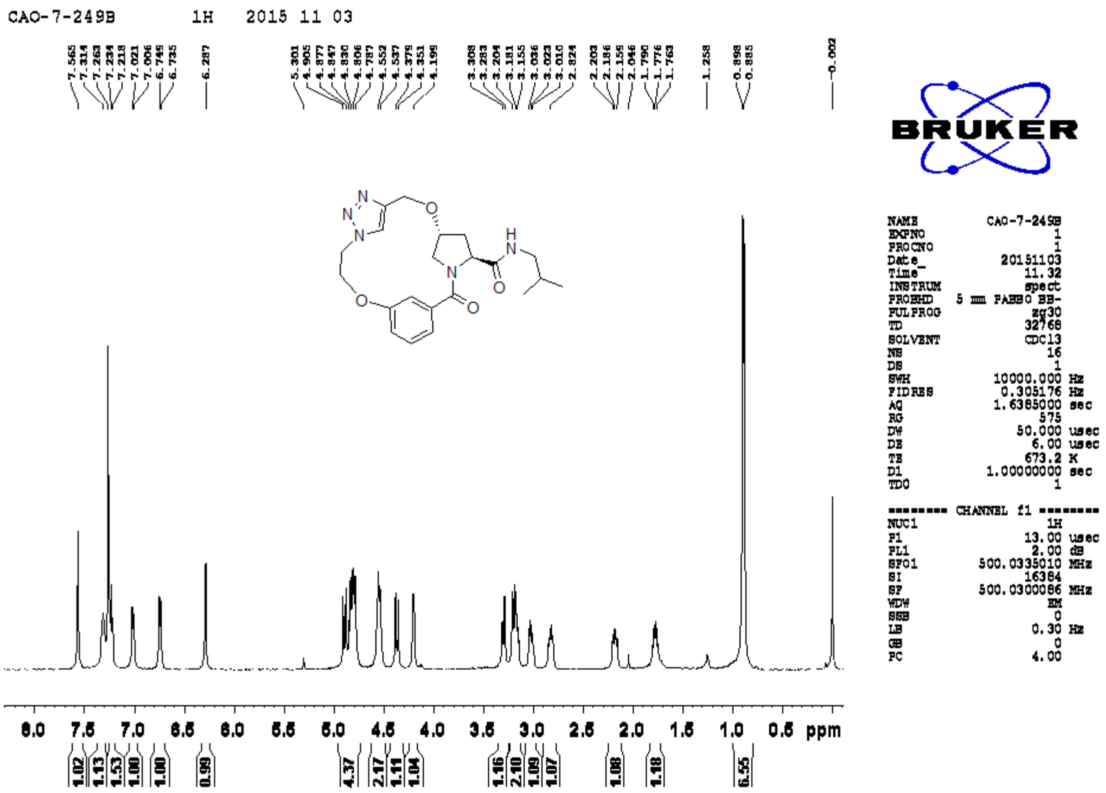

Figure S17. ^{1}H-NMR Spectrum of **25**.

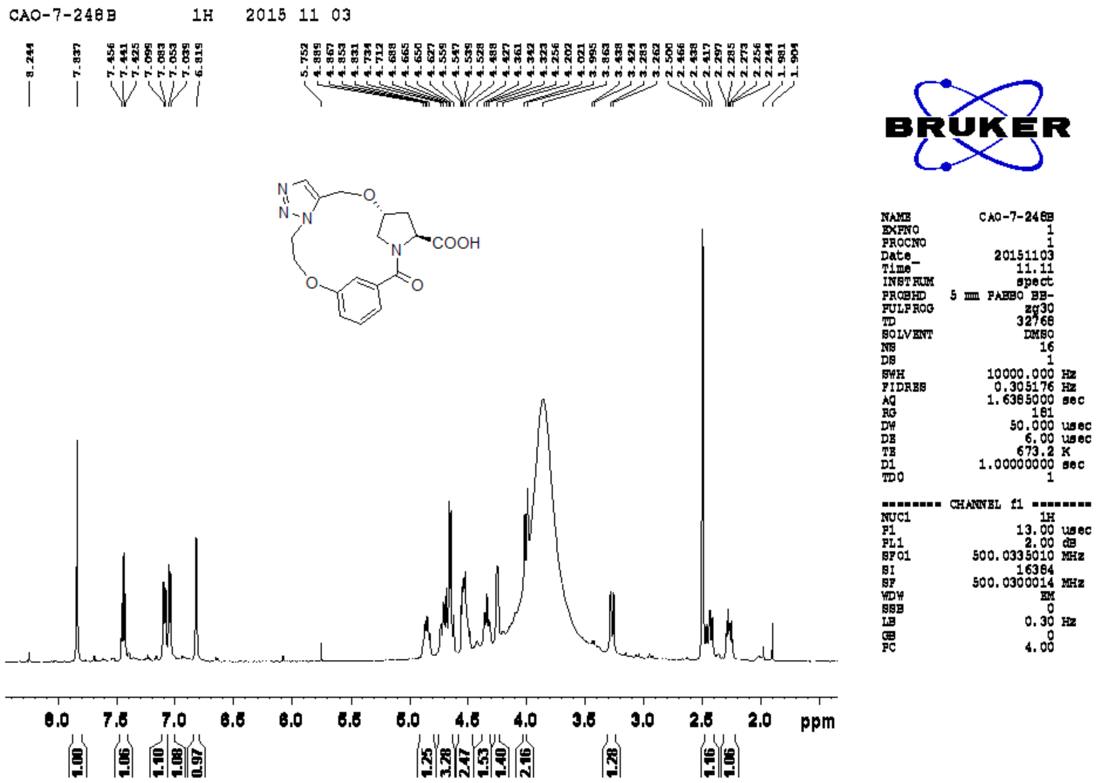

Figure S18. ^{1}H-NMR Spectrum of **26**.

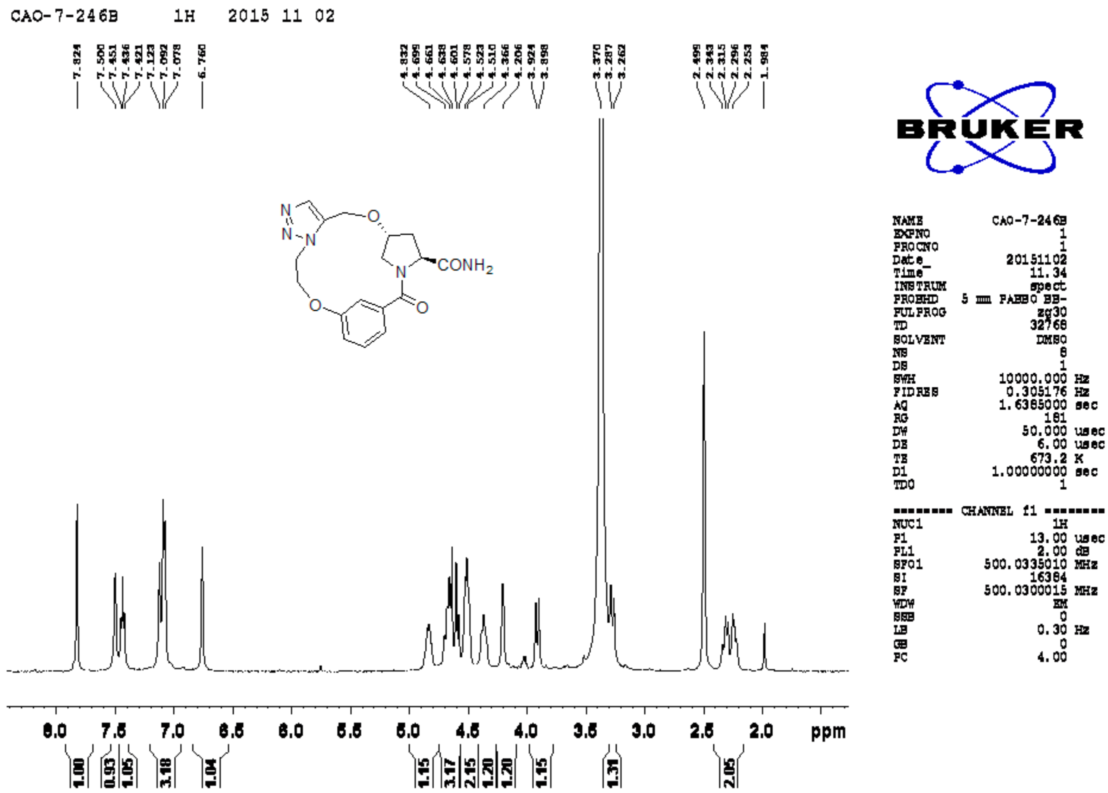

Figure S19. ^{1}H-NMR Spectrum of **27**.

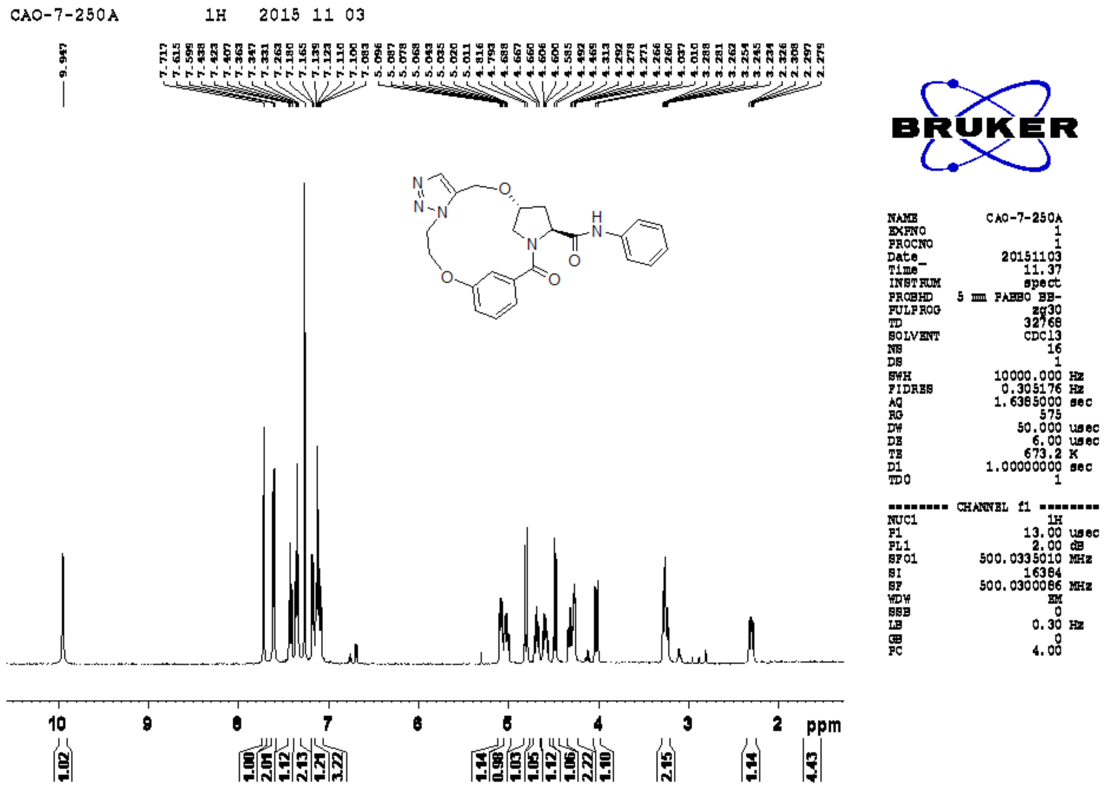

Figure S20. ^{1}H-NMR Spectrum of **28**.

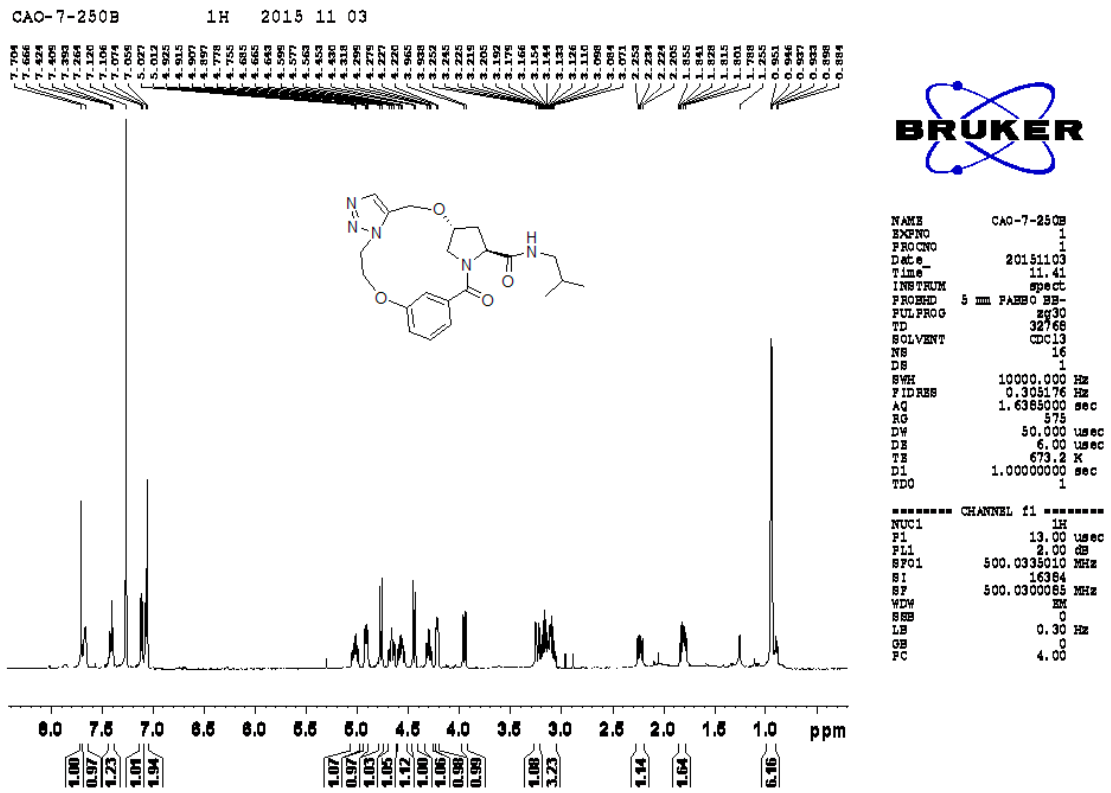

Figure S21. ^{1}H-NMR Spectrum of **29**.

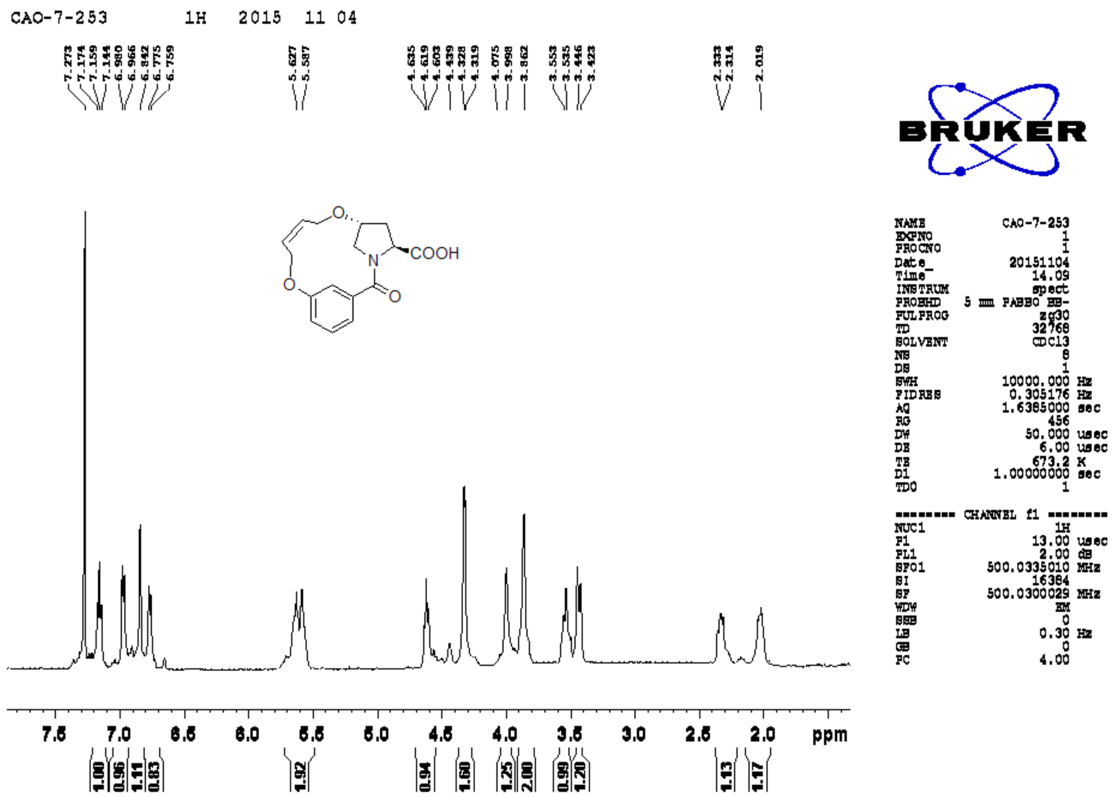

Figure S22. ^{1}H-NMR Spectrum of **30**.

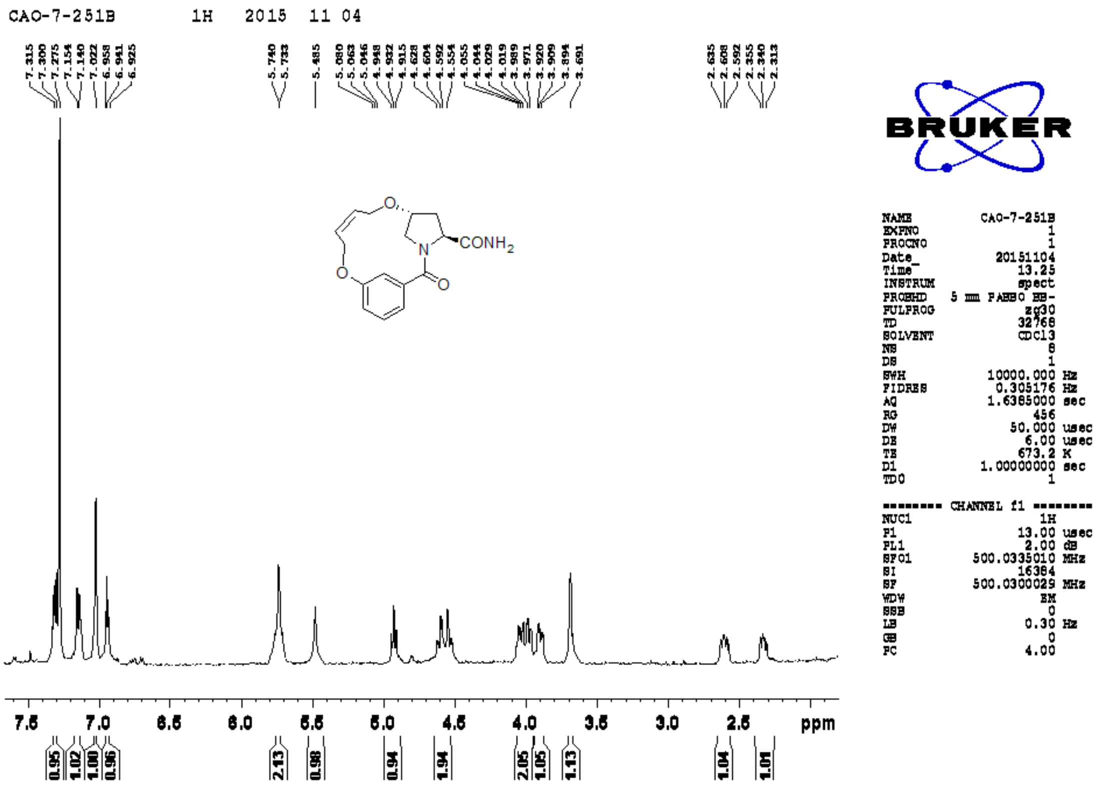

Figure S23. ^{1}H-NMR Spectrum of **31**.

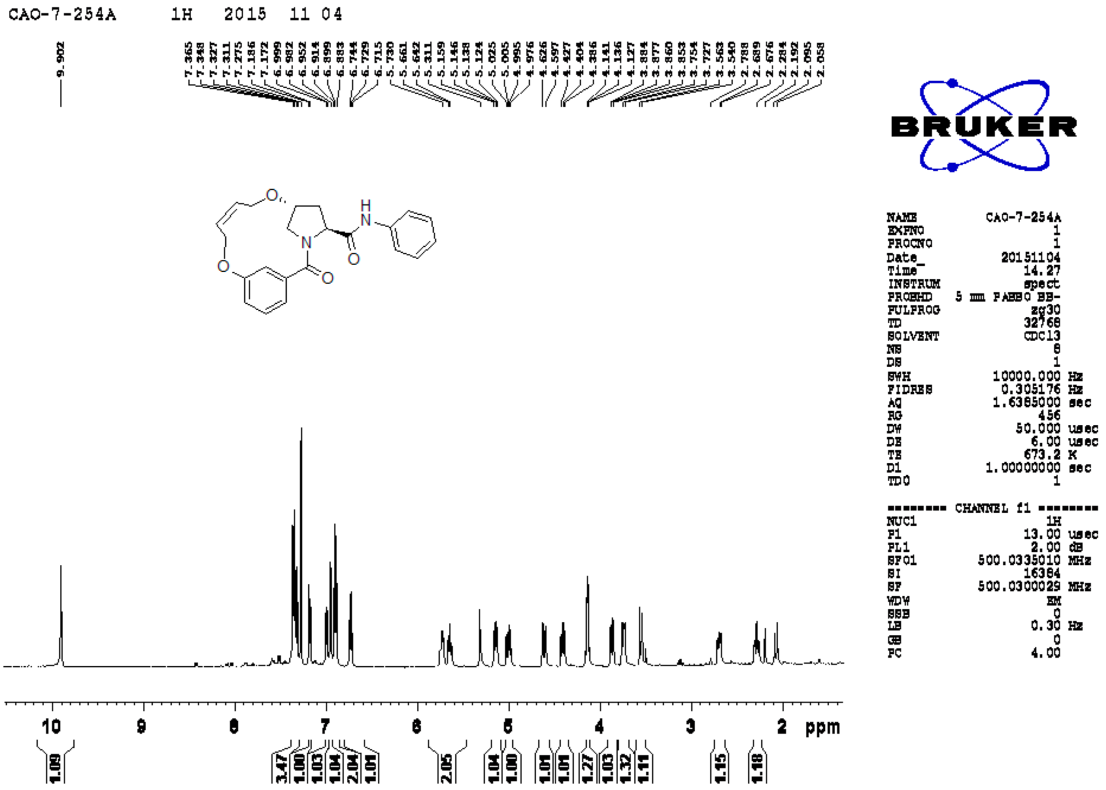

Figure S24. ^{1}H-NMR Spectrum of **32**.

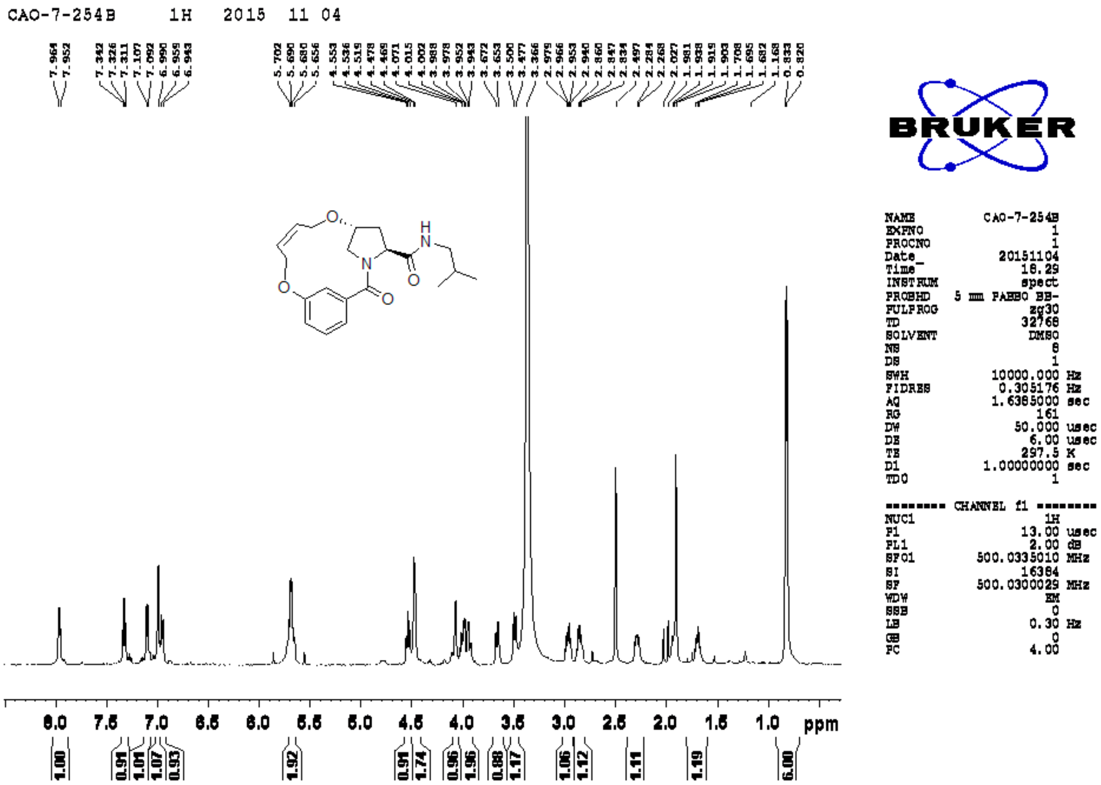

Figure S25. ^{1}H-NMR Spectrum of **33**.

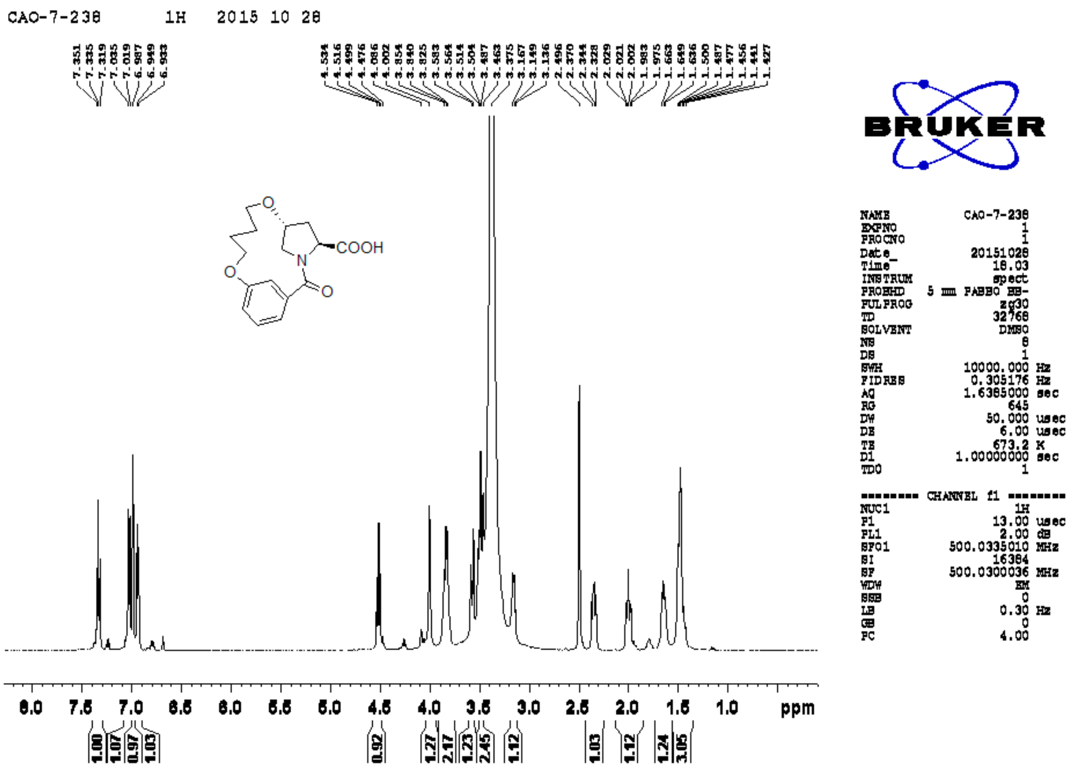

Figure S26. ^{1}H-NMR Spectrum of **34**.

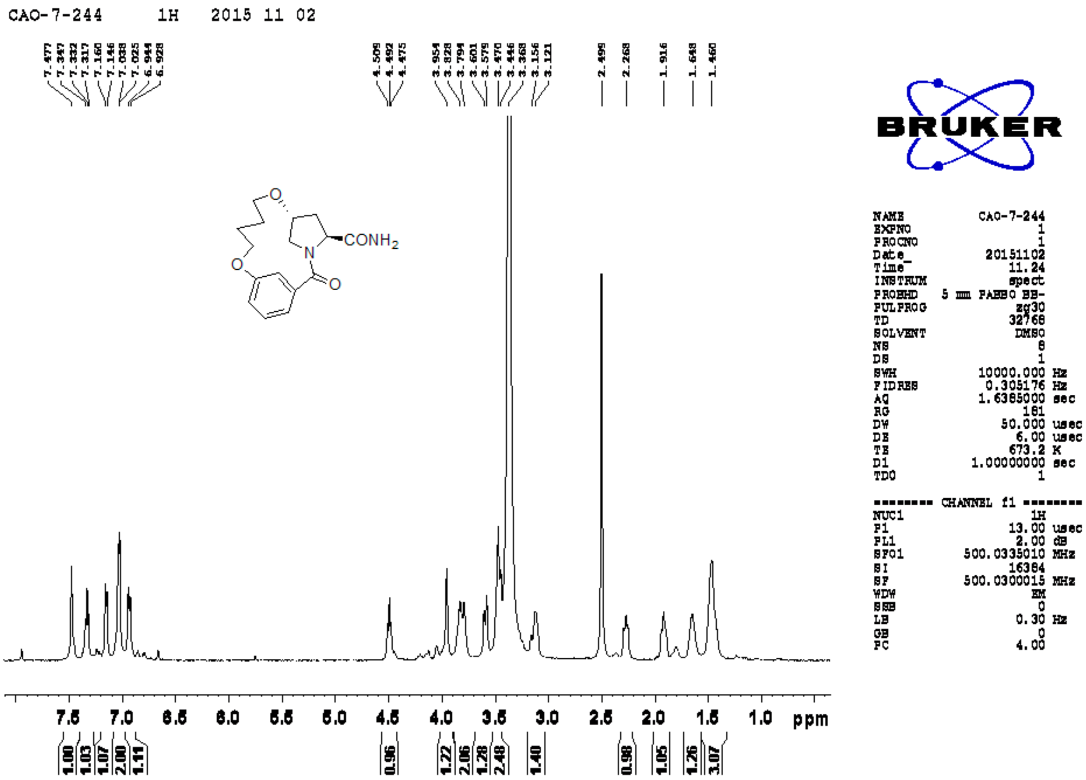

Figure S27. ^{1}H-NMR Spectrum of **35**.

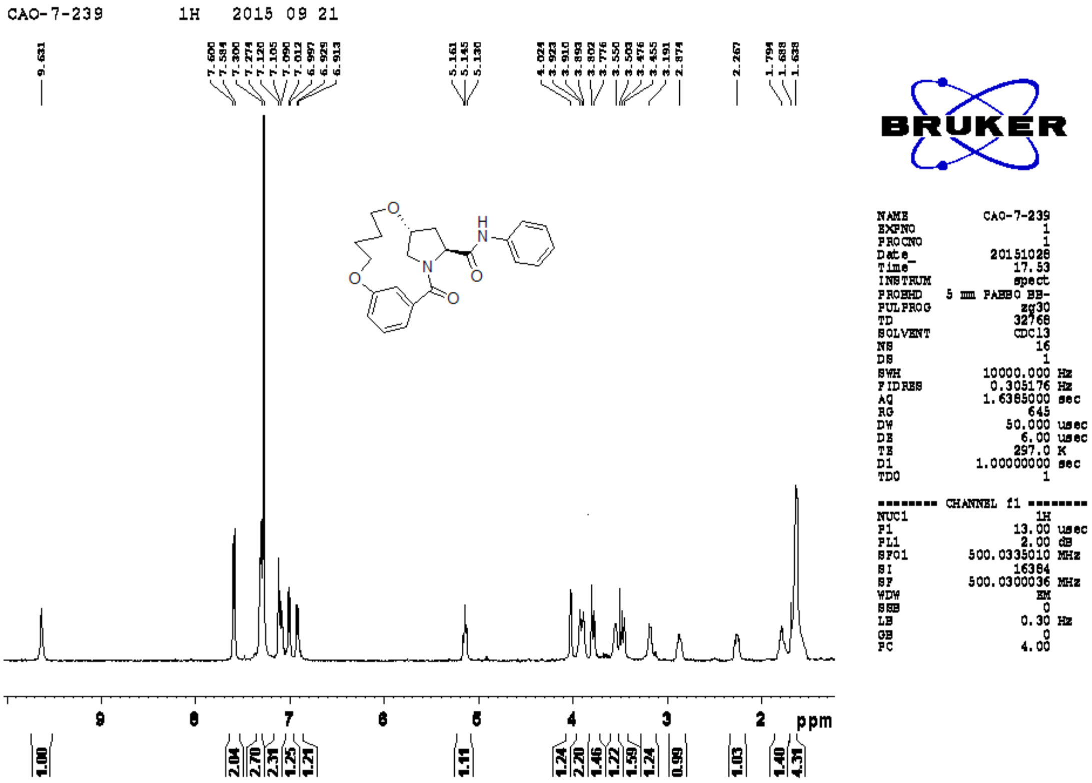

Figure S28. ^{1}H-NMR Spectrum of **36**.

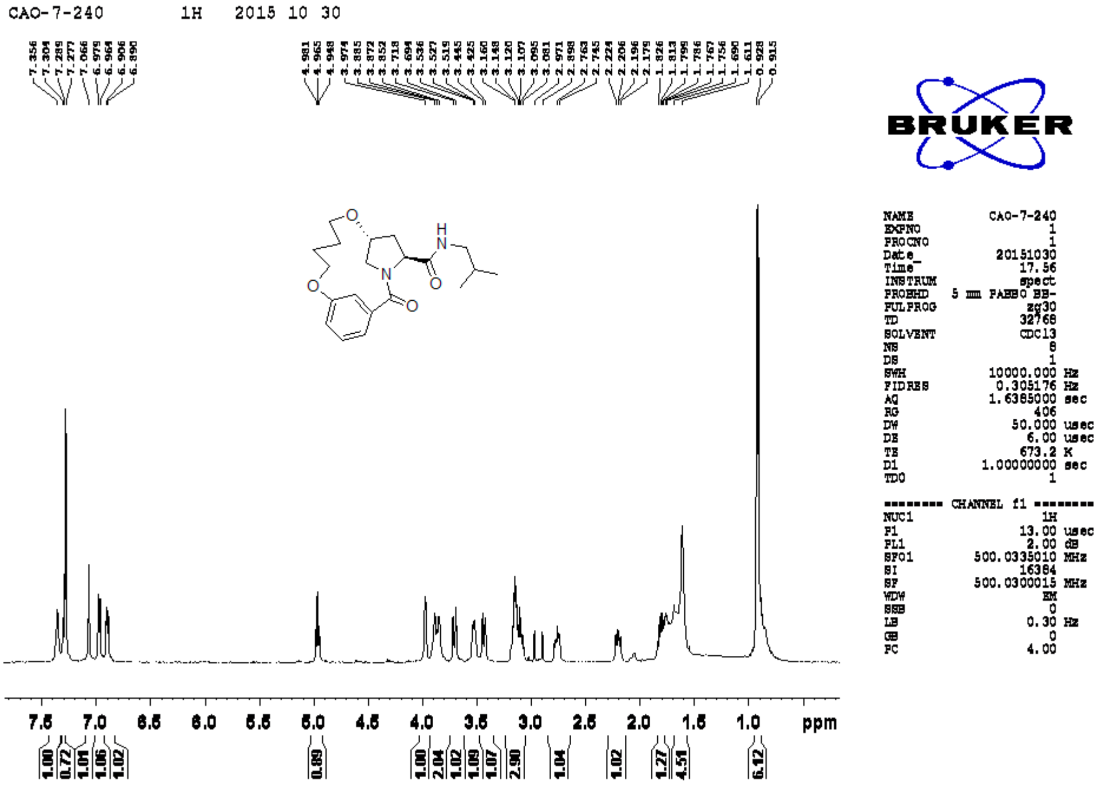

Figure S29. ^{1}H-NMR Spectrum of **37**.

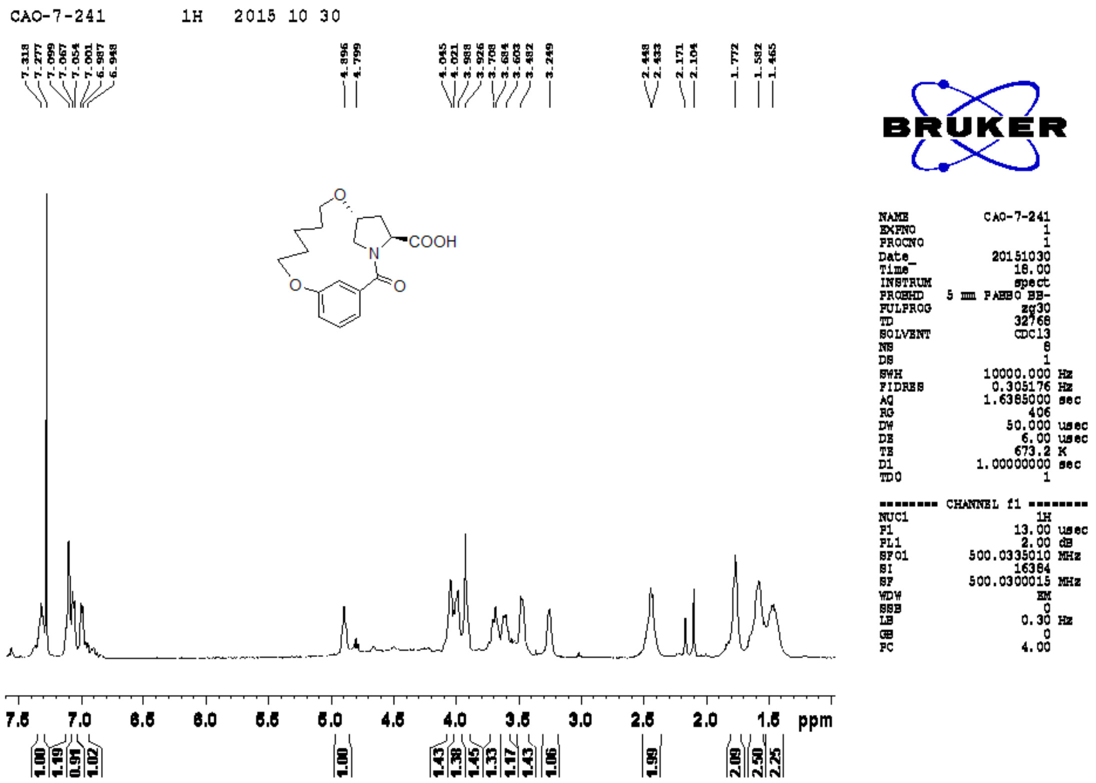

Figure S30. ^{1}H-NMR Spectrum of **38**.

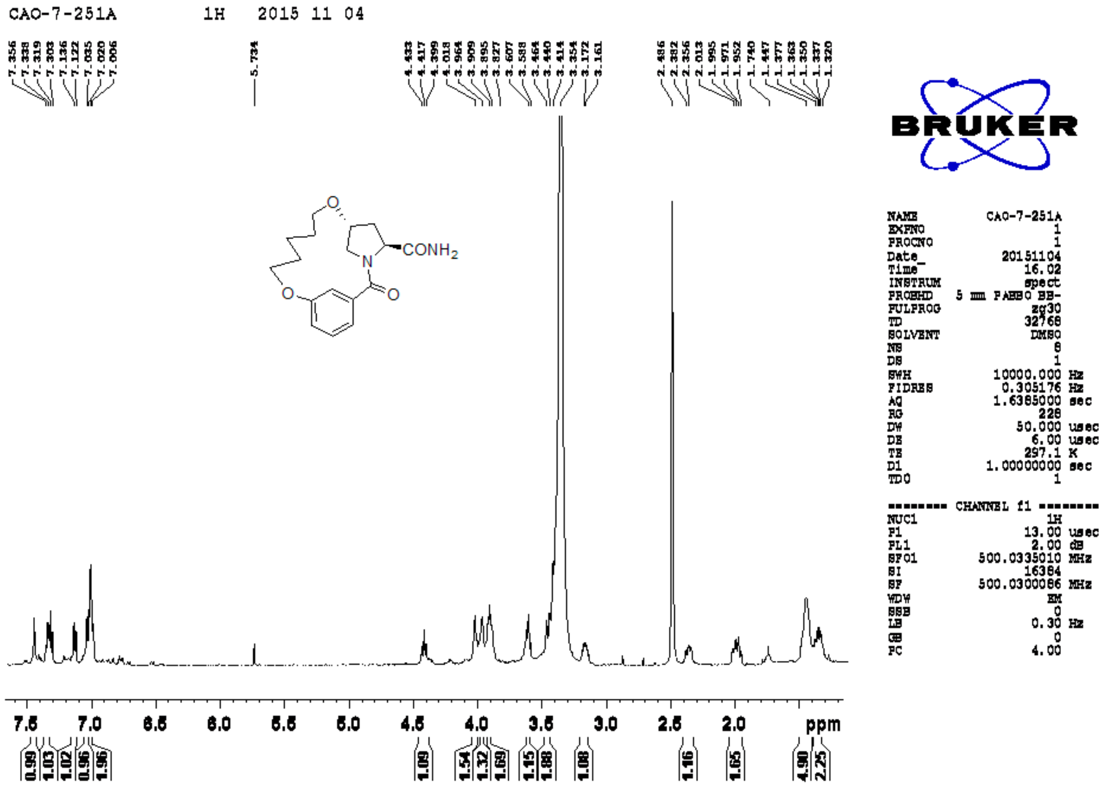

Figure S31. ^{1}H-NMR Spectrum of **39**.

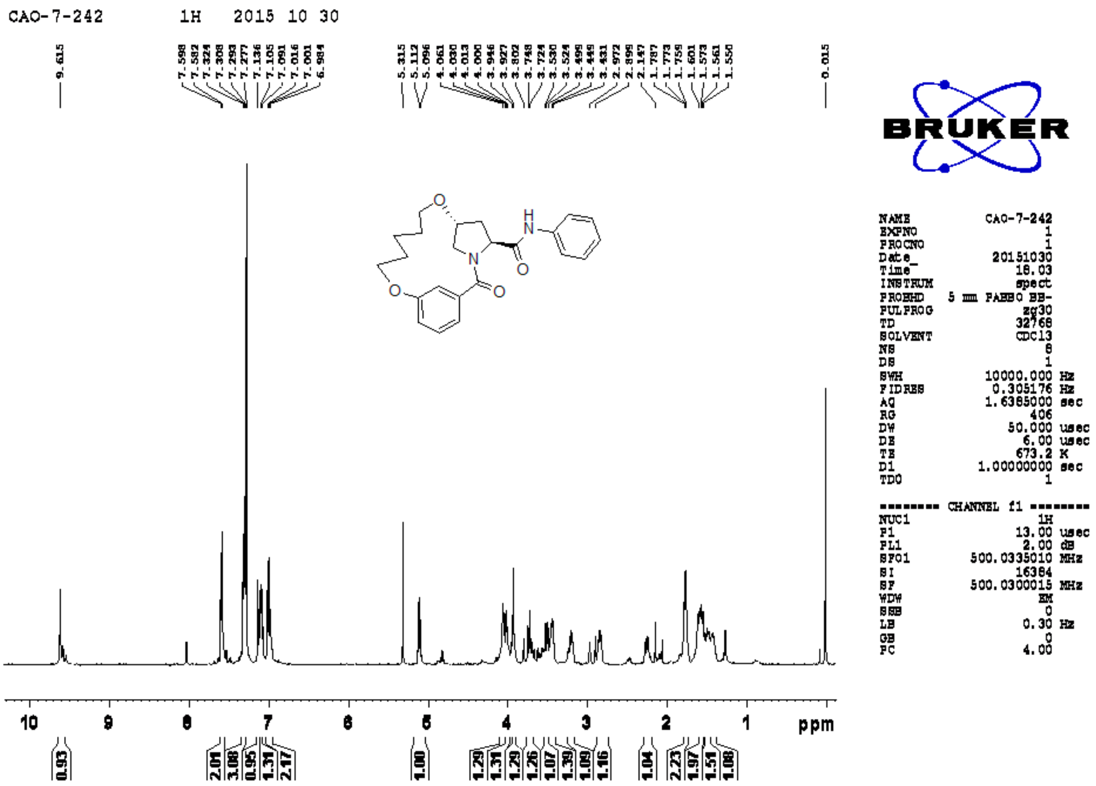

Figure S32. ^{1}H-NMR Spectrum of **40**.

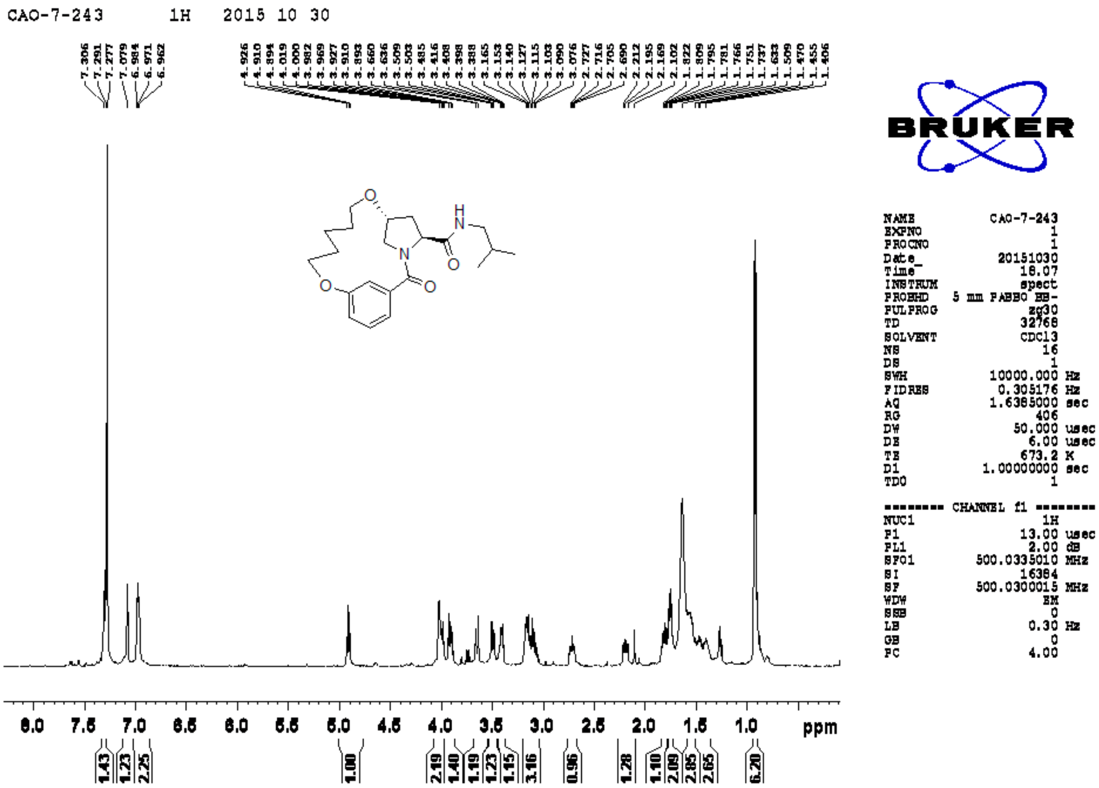

Figure S33. ^{1}H-NMR Spectrum of **41**.

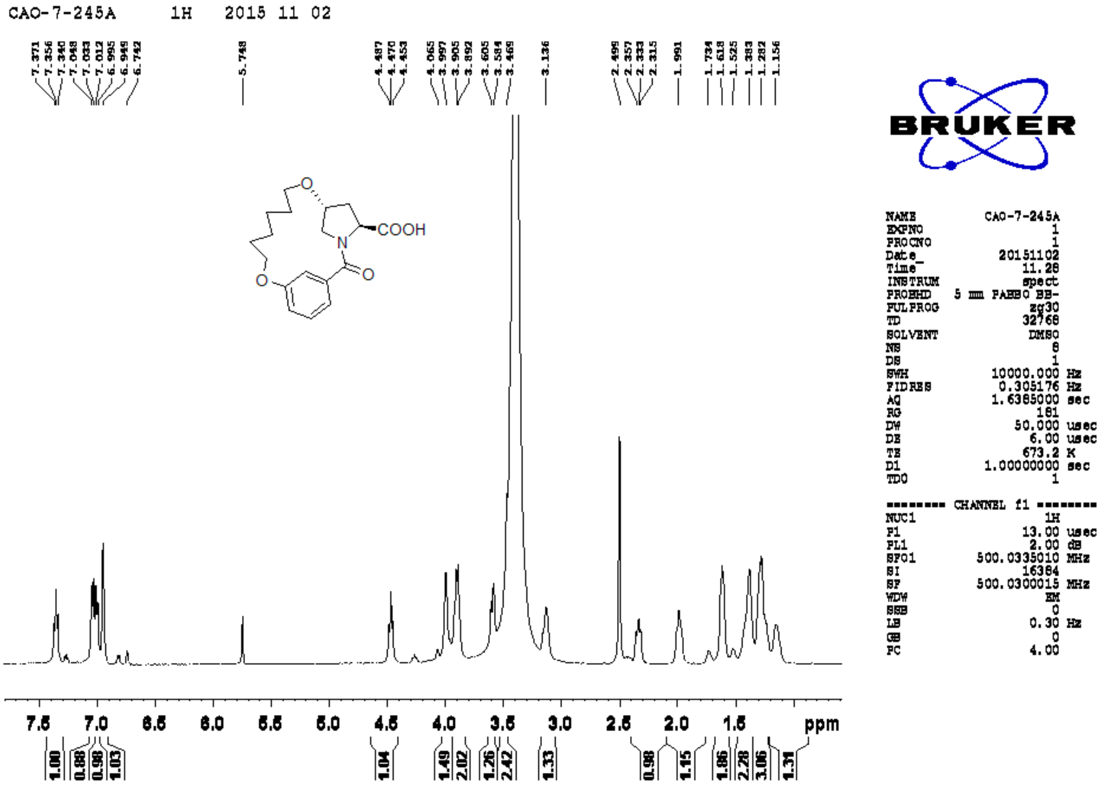

Figure S34. ^{1}H-NMR Spectrum of **42**.

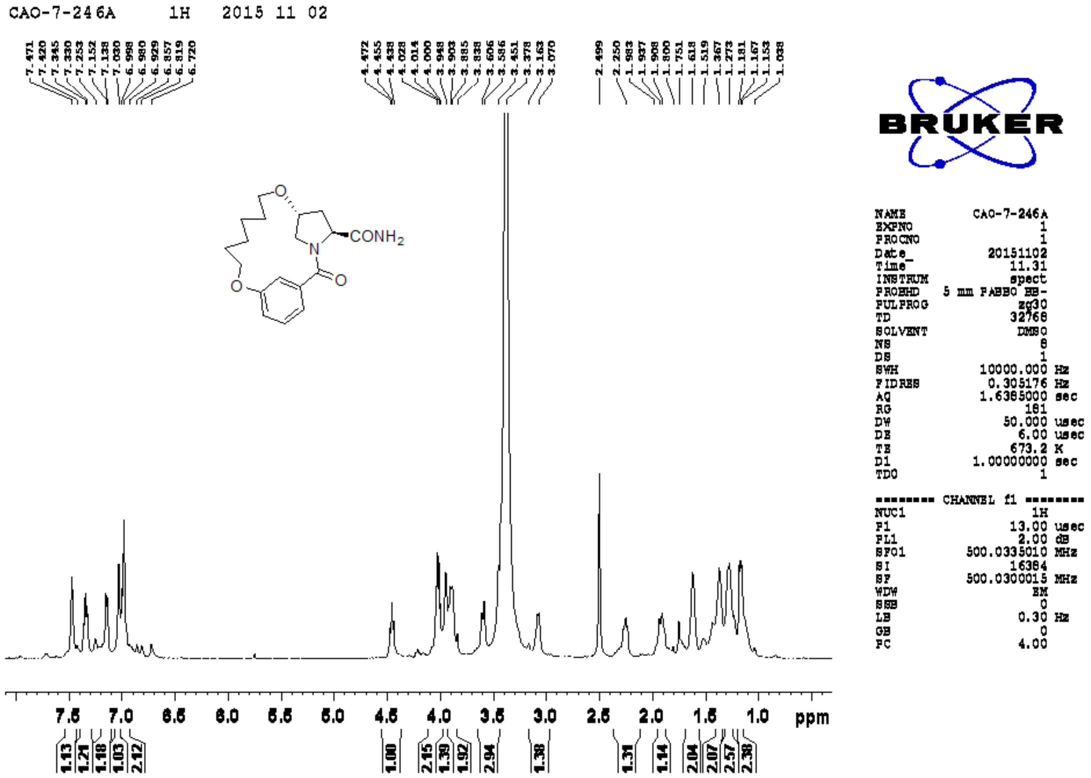

Figure S35. ^{1}H-NMR Spectrum of **43**.

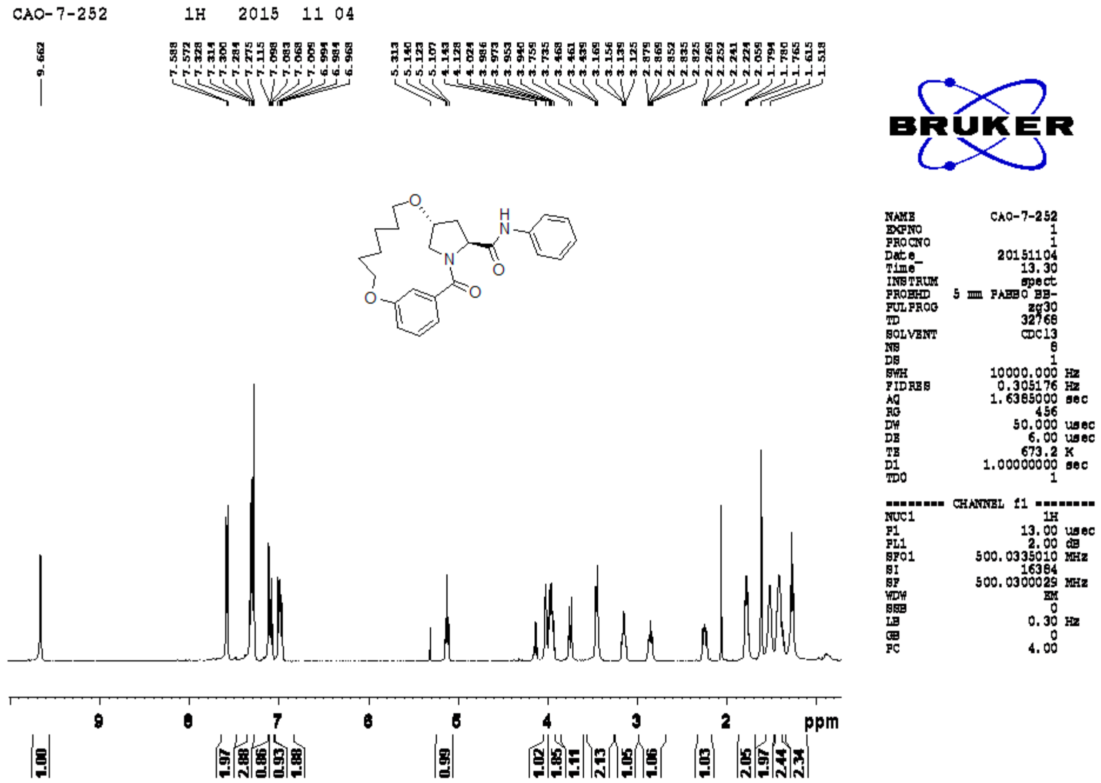

Figure S36. ^{1}H-NMR Spectrum of **44**.

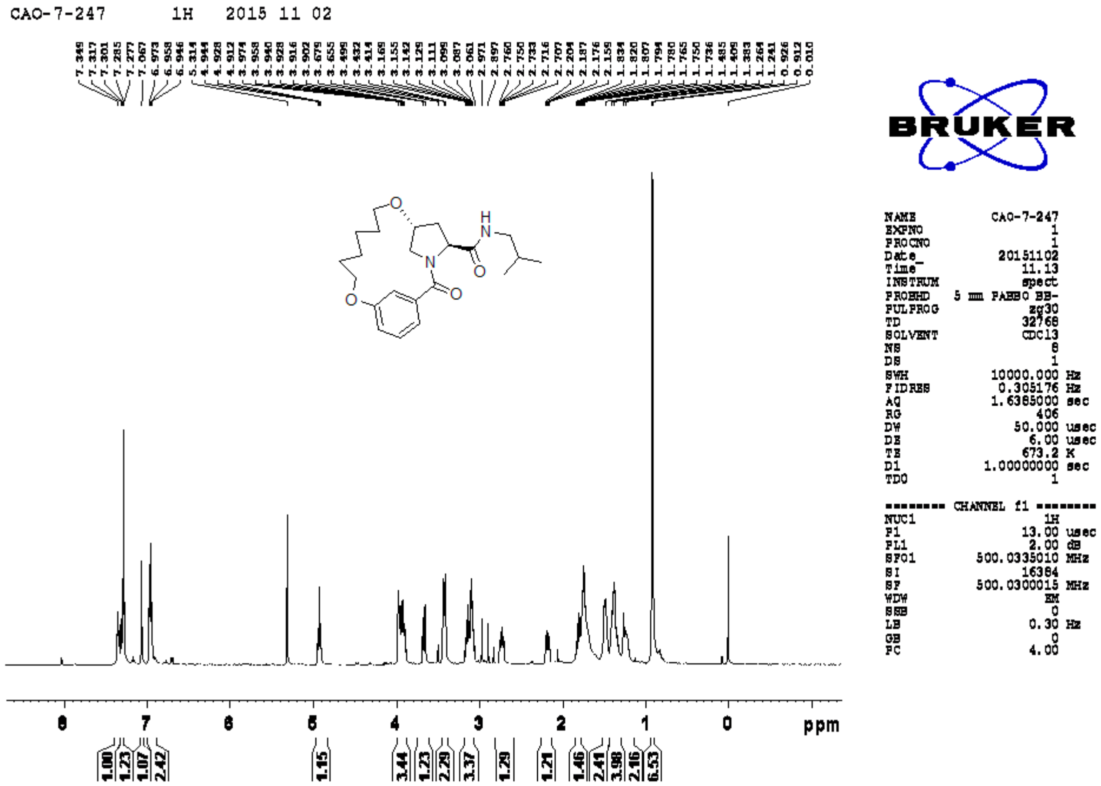

Figure S37. ^{1}H-NMR Spectrum of **45**.
